# *Acidithiobacillia* class members originating at sites within the Pacific Ring of Fire and other tectonically active locations and description of the novel genus ‘*Igneacidithiobacillus’*

**DOI:** 10.3389/fmicb.2024.1360268

**Published:** 2024-04-03

**Authors:** Dilanaz Arisan, Ana Moya-Beltrán, Camila Rojas-Villalobos, Francisco Issotta, Matías Castro, Ricardo Ulloa, Patricia A. Chiacchiarini, Beatriz Díez, Alberto J. M. Martín, Iván Ñancucheo, Alejandra Giaveno, D. Barrie Johnson, Raquel Quatrini

**Affiliations:** ^1^Facultad de Medicina y Ciencia, Universidad San Sebastián, Santiago, Chile; ^2^Centro Científico y Tecnológico de Excelencia Ciencia & Vida, Santiago, Chile; ^3^Departamento de Informática y Computación, Facultad de Ingeniería, Universidad Tecnológica Metropolitana, Santiago, Chile; ^4^Facultad de Ingeniería, Arquitectura y Diseño, Universidad San Sebastián, Santiago, Chile; ^5^Biological Sciences Faculty, Pontifical Catholic University of Chile, Santiago, Chile; ^6^Millennium Institute Center for Genome Regulation (CGR), Santiago, Chile; ^7^Instituto Milenio de Oceanografía (IMO), Universidad de Concepción, Concepción, Chile; ^8^PROBIEN (CCT Patagonia Confluencia-CONICET, UNCo), Facultad de Ingeniería, Departamento de Química, Universidad Nacional del Comahue, Neuquén, Argentina; ^9^Center for Climate and Resilience Research (CR), Santiago, Chile; ^10^Facultad de Ingeniería y Tecnología, Universidad San Sebastián, Lientur, Concepción, Chile; ^11^College of Natural Sciences, Bangor University, Bangor, United Kingdom; ^12^Faculty of Health and Life Sciences, Coventry University, Coventry, United Kingdom; ^13^Natural History Museum, London, United Kingdom

**Keywords:** *Igneacidithiobacillus*, MAG, terrestrial hydrothermal areas, Caviahue-Copahue volcanic complex, pangenome, phylogenomics, habitat

## Abstract

Recent studies have expanded the genomic contours of the *Acidithiobacillia*, highlighting important lacunae in our comprehension of the phylogenetic space occupied by certain lineages of the class. One such lineage is ‘*Igneacidithiobacillus’*, a novel genus-level taxon, represented by ‘*Igneacidithiobacillus copahuensis’* VAN18-1^T^ as its type species, along with two other uncultivated metagenome-assembled genomes (MAGs) originating from geothermally active sites across the Pacific Ring of Fire. In this study, we investigate the genetic and genomic diversity, and the distribution patterns of several uncharacterized *Acidithiobacillia* class strains and sequence clones, which are ascribed to the same 16S rRNA gene sequence clade. By digging deeper into this data and contributing to novel MAGs emerging from environmental studies in tectonically active locations, the description of this novel genus has been consolidated. Using state-of-the-art genomic taxonomy methods, we added to already recognized taxa, an additional four novel *Candidate* (*Ca.*) species, including ‘*Ca.* Igneacidithiobacillus chanchocoensis’ (mCHCt20-1^TS^), ‘*Igneacidithiobacillus siniensis’* (S30A2^T^), ‘*Ca.* Igneacidithiobacillus taupoensis’ (TVZ-G3 ^TS^), and ‘*Ca.* Igneacidithiobacillus waiarikiensis’ (TVZ-G4 ^TS^). Analysis of published data on the isolation, enrichment, cultivation, and preliminary microbiological characterization of several of these unassigned or misassigned strains, along with the type species of the genus, plus the recoverable environmental data from metagenomic studies, allowed us to identify habitat preferences of these taxa. Commonalities and lineage-specific adaptations of the seven species of the genus were derived from pangenome analysis and comparative genomic metabolic reconstruction. The findings emerging from this study lay the groundwork for further research on the ecology, evolution, and biotechnological potential of the novel genus ‘*Igneacidithiobacillus*’.

## Introduction

*Acidithiobacillia* class bacteria (Williams and Kelly, 2013) catalyze the dissimilatory oxidation of sulfur at diverse pH and temperature optima in sulfide mineral settings, being key players in the biogeochemical cycling of sulfur, iron, and other metals in mildly to highly acidic aquatic environments. Phenotypic and genotypic variability of its members are well acknowledged and have motivated several instances of revision of the taxonomy of the group (Johnson and Quatrini, 2020). Currently, the class comprises ten validated species (Boden and Hutt, 2019) and eight recently acknowledged novel species (Moya-Beltrán et al., 2021), in addition to several subspecies-level lineages (Nuñez et al., 2017; Moya-Beltrán et al., 2023). These studies have significantly expanded the genomic contours of the class and highlighted important lacunae in our comprehension of the phylogenetic space occupied by acidithiobacilli-like bacteria.

One clade requiring further study is the 16S rRNA Clade 1, which was pinpointed in a class-wise phylogenetic study of the 16S rRNA gene oligotypes (Nuñez et al., 2017). This clade encompasses two sister branches: branch 1A groups all sequenced representatives of ´*Fervidacidithiobacillus caldus*´ (formerly *Acidithiobacillus caldus*) and branch 1B/1C groups several uncharacterized strains and sequence clones originating from tectonically active thermal sites in the island of Vulcano in Italy (Norris et al., 2020), the Copahue Volcano in Argentina/Chile (Moya-Beltrán et al., 2021), and also sulfidic caves and ores in America and Asia (Ni et al., 2008; Jones et al., 2016). Culturable isolates of the clade have been recovered recently for the Caviahue-Copahue Volcanic Complex, and genomic sequences of six isolates obtained (Moya-Beltrán et al., 2021). Metagenome assembled genomes (MAGs), related to the above sequenced representatives, could be traced from different datasets, also originating from geothermally active sites in Yellowstone, Wyoming (Zhou et al., 2020a), Shi-Huang-Ping, Taiwan (Lin et al., 2015), and the Taupo Volcanic Zone in New Zealand (Sriaporn et al., 2021). Genomic indexes and phylogenomic analyses of this branch support the existence of a novel genus-level taxon, provisionally named ‘*Igneacidithiobacillus*’ for the characteristics of the origin source of the representative strains first described and assigned to the species ‘*Igneacidithiobacillus copahuensis*’ (Norris et al., 2020; Moya-Beltrán et al., 2021).

Here, we further expand the contours of this novel genus by identifying novel species ‘*Igneacidithiobacillus siniensis*’ (cultured) and *Candidate* species ‘*Ca.* Igneacidithiobacillus chanchocoensis’, ‘*Ca.*’ Igneacidithiobacillus yellowstonensis, ‘*Ca.*’ Igneacidithiobacillus taiwanensis, ‘*Ca.* Igneacidithiobacillus taupoensis’, and ‘*Ca.* Igneacidithiobacillus waiarikiensis’ and prospective sites for the isolation of culturable representatives, which is informed by the physicochemical characteristics of their preferred habitats. We also elucidate key morphophysiological and metabolic attributes of un/cultured members of the genus through comprehensive pangenome analyses and metabolic reconstructions. Insights emerging from this study underscore the applied potential of this genus in biomining and environmental biotechnologies.

## Materials and methods

### Gene and genome sequences

To discover potentially novel *Acidithiobacillia* class Clade 1 B/C bacteria, we downloaded the 16S small subunit ribosomal RNA gene sequences from GenBank (as of April 2023), showing sequence identity of >97% to the reference gene sequence recovered from the ‘*Igneacidithiobacillus copahuensis’* VAN18-1 strain (locus: HFQ13_RS04740). Recovered 16S rRNA gene sequences were oligotyped, according to Nuñez et al. (2017). Accession numbers and annotations for the dataset retained for further analysis are presented in Supplementary Table S1. Public genomes of Clade 1 B/C strains (VAN18-1: JAAXYO01; VAN18-2: JAAXYU01; VAN18-4: JAAXYS01; CV18-2: JAAXYP01; CV18-3: JAAXYQ01; BN09-2: JAAXYR01; YTS05: CP094359.1, CP094360.1; S30A2; JALQCS01) and reference strains used as outgroups and controls (`*Fervidacidithiobacillus caldus*’ ATCC 51756^T^: CP005986-CP005989; `*Ambacidithiobacillus sulfuriphilus*´ DSM 105150^T^: RIZI01 and *Thermithiobacillus tepidarius* DSM 3134^T^: AUIS010) were obtained from NCBI^1^ on April 2023.

### Metagenome-assembled genome recovery, assembly, and refining

We recovered the MAGs assigned by Moya-Beltrán et al. (Moya-Beltrán et al., 2021) to ‘*Ca.* Igneacidithiobacillus yellowstonensis’ (Spst-908: formerly DTMS01, now SRR7540054) and ‘*Ca.* Igneacidithiobacillus taiwanensis’ (UBA2486: DDOU01), as well as three novel MAGs of interest identified as ‘*Igneacidithiobacillus’* representatives in hot spring metagenomes of the Taupo Volcanic Zone (BioProject: PRJNA644733) with the following NCBI whole genome sequence identifiers: TVZ_G2, JAEPKW01; TVZ_G3, JAEPKX01 and TVZ_G4, JAEPKY01.

MAG sequence mCHCt20-1 (JAWNZB01), included in this study, was recovered from environmental sequencing data generated for a slurry sample collected in 2020 from a hot pool at ChanchoCó (BioBio region, Chile) designated CHCt (−37.818611 S, −71.163611 W; 1,798 m.a.s.l.; 38.9–56°C; pH 5.8–7.0). Sample manipulation for DNA sequencing and sequence data pre-processing and assembly were performed as detailed in the study by Degli Esposti et al. (2023). Contigs larger than 1,000 bp were grouped into genome bins by MaxBin2 v2.2.7 (Wu et al., 2016), Metabat2 v2 (Kang et al., 2019), and CONCOCT v1.1.0 (Alneberg et al., 2014) using the default parameters. Conserved marker genes were evaluated using CheckM v1.1.3 (Parks et al., 2015), CheckM2 v0.1.2 (Chklovski et al., 2023), and QUAST toolkit (Mikheenko et al., 2018). MAG assembly statistics were used to classify MAGs according to accepted quality standards (Bowers et al., 2018). Small-subunit rRNA sequences in contigs were identified using Barrnap (Seeman, 2017).

### Genomic taxonomy analyses

To evaluate the taxonomic assignment of strains and MAGs within the assembled set, genomic indexes based on amino acid and nucleotide sequences data were derived from the available genomic information. The average amino acid identity (AAI) was calculated using the CompareM program^2^ and the aai.rb implementation from the Kostas Lab (Rodriguez-R and Konstantinidis, 2016; https://github.com/lmrodriguezr/enveomics; commit signature: fae592f) and run using the default parameters. Genus- and species-level AAI% cutoff thresholds used were < 62–70% and > 95.38, respectively (Richter and Rosselló-Móra, 2009). The average nucleotide identity based on Blast (ANIb) or Mummer (ANIm) as alignment algorithm, were calculated for all possible genome-MAGs pairs using a Python module implemented by Pritchard et al. (2016), which was available at https://github.com/widdowquinn/pyani. The *in silico* DNA–DNA hybridization index (dDDH) was assessed using the Genome-to-Genome Distance Calculator, and the cutoff values defined by Meier-Kolthoff et al. (2013, 2014), available at http://ggdc.dsmz.de.

### Gene calling, annotation, and clustering

Gene calling and annotation were performed using the NCBI Prokaryotic Genome Annotation Pipeline (PGAP, Tatusova et al., 2016; Haft et al., 2018). Low-quality MAGs (DDOU01 and DTMS01) were annotated through the Rapid Annotation Subsystem Technology pipeline (RAST, Aziz et al., 2008). Functional assignments were validated against the Conserved Domain Database v.3.16 (CDD, Marchler-Bauer et al., 2017) using CDsearch (Marchler-Bauer and Bryant, 2004) and hhsearch (Fidler et al., 2016) with default parameters. All predicted proteins were also analyzed against the profiles stored in the KEGG and COG databases (updated on July 2023) using the SqueezeMeta pipeline (Tamames and Puente-Sánchez, 2019). Annotated proteins were clustered by identity (threshold of 50% and default parameters) using Usearch v1.2.22 (Edgar, 2010). Protein alignments were constructed using the MAFFT v7.123 program (Katoh and Standley, 2013). Selected clusters and proteins were re-annotated and curated manually.

### Comparative genomics methods

Gene orthology was calculated by the GET_HOMOLOGUES software package v3.3.2 (Contreras-Moreira and Vinuesa, 2013) using COGtriangles v2.1 as clustering algorithm (Kristensen et al., 2010), to recover Protein Families (PFs). BLAST pairwise alignment cutoffs were set at 75% coverage and, E-values were set at 10E-5. Gene copy numbers per PF and genome/MAG were scored by in-house Perl/Phyton scripts using E-value cutoff of 10E-5 and identity cutoff of 60%, to discriminate orthologs from paralogs. The results were curated based on genomic context analysis and manually revised alignments, when appropriate. Pan-genome metrics, including the size of core, flexible, and exclusive gene complements, were calculated by Tettelin et al. (2005). Phyletic patterns were constructed by scoring the presence/absence of representatives of each PF in each genome/MAG using parse_pangenome_matrix.pl. and other in-house scripts. Data analysis and visualization were conducted using the R package tidyverse v1.3.0.

### Phylogenetic analysis of 16S rRNA, Fis and core proteins

Small subunit ribosomal RNA gene sequences and different sets of protein families of ecophysiological/evolutionary interest (Fis; conserved core proteins) were aligned using MAFFT v7.310 software with the L-INS-I method (Katoh and Standley, 2013). The 16S rRNA gene alignments were trimmed and masked (>50%) using trimAl v1.2 (Capella-Gutiérrez et al., 2009) and checked manually. Protein alignments were generated individually for each protein family with MAFFT settings *maxiterate* 1,000 and *localpair,* and the resulting alignments were trimmed using trimAl v1.2 with gap threshold of 0.5 and further refined manually. Phylogenetic trees were generated by neighbor-joining (NJ), maximum likelihood (ML), and Bayesian inference (BI) analysis to produce alternative phylogenies (Moya-Beltrán et al., 2021). The 16S rRNA gene NJ algorithm was implemented in quicktree (Howe et al., 2002), with the following settings *upgma* and *kimura,* and bootstrap values were calculated over 10,000 iterations. The ML tree was constructed using PhyML v3.0 (Guindon et al., 2010), with the following settings: General Time Reversible model (GTR, Tamura and Nei, 1993) was used as substitution model, PhyML estimated the transition/transversion ratio, the proportion of invariant nucleotides, and a discrete gamma approximation with *k* = 4 and 10,000 bootstrap replicates. The topology of the tree and the length of the branches were optimized by PhyML using Nearest Neighbor Interchange and Subtree Pruning and Regrafting. The Bayesian analysis tree was built with MrBayes v3.2.7 (Huelsenbeck and Ronquist, 2001) and run for 3,000,000 generations, saving trees every 100 generations. Posterior probabilities were calculated after discarding the first 30% of trees. Maximum likelihood Fis and core protein trees were reconstructed by PhyML v3.0 using Le Gascuel (LG) and Whelan and Goldman (WAG) amino acid substitution models, respectively, and tree topology optimization was performed using SMS v1.8.4 with 10,000 bootstrap replicates. Bayesian phylogenetic analysis was conducted on the core and Fis protein sets using MrBayes v3.2.7. Alignments were trimmed and manually curated before phylogenetic inference. BI trees were constructed using the Whelan and Goldman (WAG) and Jones-Taylor-Thornton (JTT) amino acid substitution models. The settings used were as follows: pRSET was used to select the amino acid substitution model, with *a posteriori* probability deviation equal to or near zero and lset for gamma-distributed rate variation. Each analysis was executed with MCMC to set the number of generations to 10,000, with a sample frequency of 100 generations.

### Statistical analysis of the metadata

Pandas (Reback et al., 2020) and Scikit-learn (Pedregosa et al., 2011) python libraries were employed for metadata-based clustering of all strains studied (see first section of Results). K-means was performed using cluster purity as the error function chosen to select the best number of clusters, altering only the number of clusters. A Random Forest algorithm was trained to predict the most common lineages. Metadata features were ranked by their ability to separate lineages. K-means was used with the top scoring features and the same purity selection protocol. In addition, the chi-square test was also tried to select top-ranked strain attributes. Both selection criteria were applied to those features available for all strains and, in a second run, to all features annotated for at least 100 strains. Visual inspection of the clustering, before and after selecting features, was performed with Seaborn clustermaps (Waskom, 2021).

### Data visualization and manipulation

Summary statistics and figures were computed using the R packages: gdata v2.18.0, dplyr v1.0.2, plotly v4.9.0, ggplot2 v3.2.1, scales v1.0, RColorBrewer v1.1.2, readr v1.2.1, and Rbase v3.6.1 implemented in Rstudio v1.2.50001 (RStudio Team, 2020). Gene cluster comparisons were obtained with clinker & clustermap (Gilchrist and Chooi, 2021). Improvement in vectorial figures was made using Inkscape v1.3.2.^3^ FigTree v1.4.4 was used for tree visualization and manipulation.^4^

## Results and discussion

### *Acidithiobacillia* clade 1B/1C strains and clones harbor sequence variability

**Figure 1:**
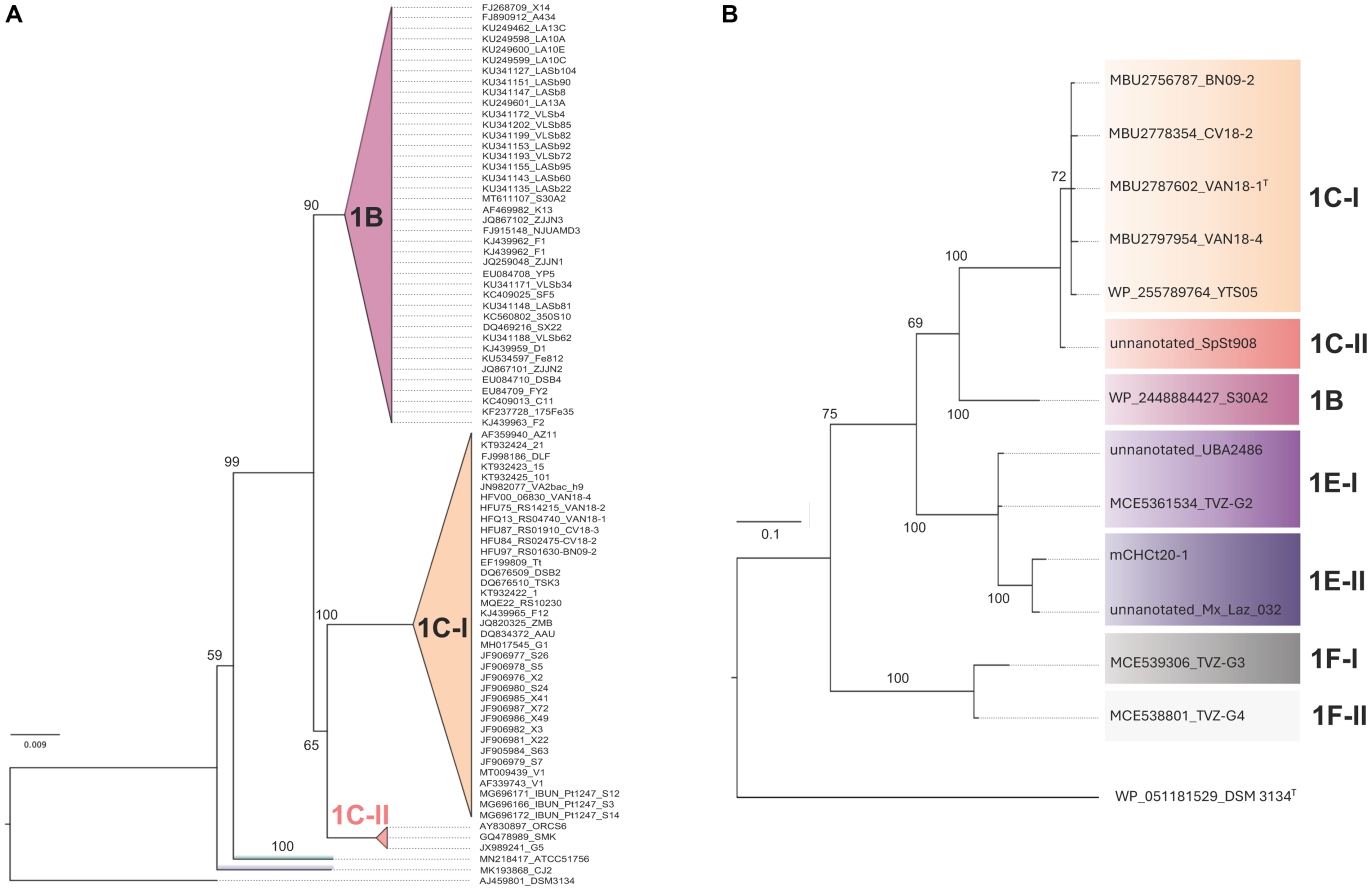
‘*Igneacidithiobacillus*’ consensus phylogenetic trees built using single marker genes. **(A)** Neighbor-joining (NJ) phylogenetic tree of 16S rRNA gene sequences assigned to ‘*Igneacidithiobacillus*’ based on an alignment encompassing 1,272 bp of the full 16S rRNA gene sequence, with 223 variable sites and 82 parsimony informative sites (see also Supplementary Figure S1A). **(B)** Bayesian inference (BI) phylogenetic tree of the Fis protein sequences assigned to ‘*Igneacidithiobacillus*’ based on an alignment encompassing 99 aa, with 55 variable sites and 43 parsimony informative sites (see also Supplementary Figure S1B). Both trees were rooted with the ortholog of *Thermithiobacillus tepidarius* DSM 3134^T^. The 16S rRNA tree included orthologs of ‘*Fervidacidithiobacillus caldus’* representative strains (clade 1A) to further resolve the branching pattern.

To assess variability of the Clade 1B/C phylotypes in the sampled and sequenced space, a comprehensive phylogenetic tree was constructed with a set of 82 16S rRNA gene sequences recovered from GenBank (April, 2023; Supplementary Table S1). The genes analyzed include orthologs of the small ribosomal subunit 16S rRNA recovered from genomes and MAGs, which are tentatively assigned to ‘*Igneacidithiobacillus*’, when available. The neighbor-joining (NJ) phylogenetic tree built with this dataset is shown in Figure 1A. The alignment encompassed 1,272 bp of the full 16S rRNA gene sequence, with 223 variable sites and 82 parsimony informative sites (see also Supplementary Figure S1). The NJ tree obtained and rooted with the 16S rRNA gene of *Thermithiobacillus tepidarius* DSM 3134^T^, showed a clear separation of all Clade 1 sequenced representatives from three distinctive subclades, one of which groups exclusively ‘*Fervidacidithiobacillus caldus’* strains (clade 1A, represented by ATCC 51756^T^). The other two sister subclades share a more recent common ancestor, each encompassing approximately half of the sequences involved in the analysis. Clade 1B includes strains originating in sulfidic caves from Mexico and sulfide ores from Colombia and China (Supplementary Table S2); in turn, Clade 1C includes strains from geothermal sites across the world (e.g., VAN18-1 from the Copahue Volcano, Argentina-Chile and V1 from Vulcano, Italy). In addition, sister subclades with diverse node depth became apparent in both branches of the tree, pertaining to ‘*Igneacidithiobacillus*’ (Table 1). Among these 16S rRNA sequences, 62% pertained to sequence clones from uncultured representatives of the class, 13.3% of which came from sites lacking verifiable metadata. Approximately 38% corresponded to isolates, of which only 8 strains (17.4% of cultured) had drafts or fully sequenced genomes (Supplementary Table S1). Inadvertently, some unclassified or misclassified strains of this group have been acknowledged for the biotechnological potential in applications ranging from the removal of sulfur-containing malodorous gases to chalcopyrite bioleaching (Lee et al., 2006; Kumar et al., 2008; Feng et al., 2012; Zhu et al., 2014; Li et al., 2018).

**Table 1:** Lineages of ‘*Igneacidithiobacillus*’ inferred from single gene markers phylogenetic analysis.

**Strain**	**16S rRNA (Fis) Clade**	**Source**	**Site of origin**
VAN18-1,2,4	1C-I (1C-I)	Hot spring water	Vertiente del Agrio Norte (VAN), Caviahue-Copahue Volcanic Complex, Argentina
CV18-2,3	1C-I (1C-I)	Acidic volcanic watershed	Cabellera de la Virgen (CV), Caviahue-Copahue Volcanic Complex, Argentina
BN09-2	1C-I (1C-I)	Hot spring slurry	Baño 9 (BN), Caviahue-Copahue Volcanic Complex, Argentina
YTS05	1C-I (1C-I)	Acid water from a tailing pit	Yantai, China
SpSt-908	ND (1C-II)	Hot spring sediment	Yellowstone, United States
S30A2	1B (1B)	Acid mine sediment	ND Acid Mine Drainage, China
UBA2468	ND (1E_I)	Hot spring water	Shi-Huang-Ping, Taiwan
TVZ-G2	ND (1E-I)	Siliceous digitate sinter	Tikitere (TIK2), Taupo Volcanic Zone, New Zealand
mCHCt20-1	ND (1E_II)	Hot spring slurry	ChanchoCó (CHC), Caviahue-Copahue Volcanic Complex, Chile
TVZ-G3	ND (1F-I)	Siliceous digitate sinter	Tikitere (TIK2), Taupo Volcanic Zone, New Zealand
TVZ-G4	ND (1F-II)	Hot spring sediment	Tikitere (ARC_TD2), Taupo Volcanic Zone, New Zealand

16S rRNA and Fis clades derived from the phylogenetic analysis presented in Figure 1.

Given that certain MAGs assigned to ‘*Igneacidithiobacillus*’ (Moya-Beltrán et al., 2021) or inferred to pertain to the genus (TVZ_G2-G4; Sriaporn et al., 2021) lack the 16S rRNA gene marker in their current assemblies, we analyzed the tree for the *fis* gene product, which has recently been proven to be phylogenetically informative for the *Acidithiobacillia* class (Beard et al., 2023). The Fis ML tree produced with proteins recovered from the protein repository, sequenced genomes, MAGs tentatively assigned to ‘*Igneacidithiobacillus*’ (Supplementary Table S3), and similarly rooted (*T. tepidarius* DSM 3431^T^), allowed us to further resolve the branching pattern (Figure 1B). In this case, Clade 1B and 1C strains appeared in sister clades encompassing an unclassified species (1B, represented by strain S30A2) and proposed ‘*Igneacidithiobacillus copahuensis’* representatives (1C-I, 7 strains) and ‘*Ca.* Igneacidithiobacillus yellowstonensis’ single MAG (1C-II, SpSt-908). ‘*Ca.* Igneacidithiobacillus taiwanensis’ single MAG (UBA2468) was branched apart from the clade 1B/C together with the TVZ_G2 MAG from Tikitere in New Zealand, reported by Sriaporn et al. (2021) (novel clade 1E-I), and a MAG from ChanchoCó in Chile, mCHCt20-1 (novel clade 1E-II), contributed in this study. One additional clade, more ancestral in origin, accommodates the TVZ_G3 (novel clade 1F-I) and the TVZ_G4 (novel clade 1F-II) MAGs from Tikitere in New Zealand.

Collectively, these results indicate that additional taxa pertaining or related to ‘*Igneacidithiobacillus*’ exist in the currently sampled dataset, further supporting the recognition of this taxonomic unit as a genus of the *Acidithiobacillia* class. If the novel Fis clades (1E-I, 1E-II, 1F-I, and 1F-II) represent species of the genus ‘*Igneacidithiobacillus*’, or additional genus-level rank taxa, remains untested.

### Genomic indexes convey Fis clades as novel species of ‘*Igneacidithiobacillus*’

**Figure 2:**
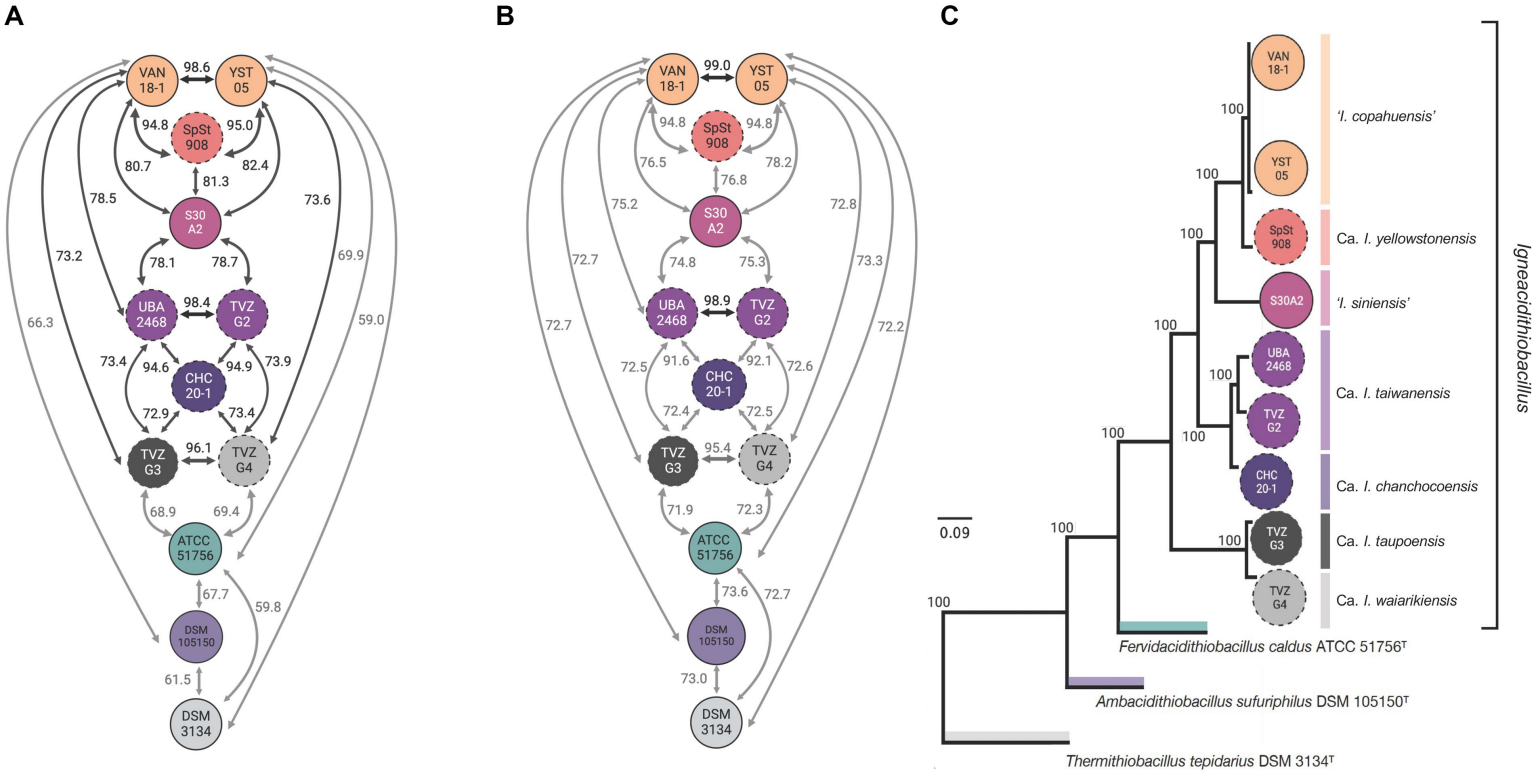
Relatedness of ‘*Igneacidithiobacillus*’ novel *Candidate* lineages inferred from whole-genome relatedness indexes based on amino acid and nucleotide sequence identity pairwise comparisons and phylogenomic analysis. **(A)** Amino acid identity values (Supplementary Table S3A) calculated with CompareM (AAI_CM_; https://github.com/dparks1134/CompareM). **(B)** Average nucleotide identity (ANIb) values (Supplementary Table S3B) calculated with the BLAST alignment algorithm. Acknowledged cutoff values for genus (% AAI > 70%, Richter and Rosselló-Móra, 2009) and species (% ANI >96%, Richter and Rosselló-Móra, 2009; Pritchard et al., 2016). **(C)** Bayesian inference (BI) tree obtained with MrBayes v3.2.7 (Huelsenbeck and Ronquist, 2001) using 87 conserved single-copy proteins common to *Acidithiobacillia* class genomes (Moya-Beltrán et al., 2021). The multiprotein phylogenetic tree was constructed with a concatenate of 8 universally conserved ribosomal proteins and 79 other phylogenetically informative housekeeping class-core marker genes encompassing 20,789 aa with 11,231 variable sites and 6,739 parsimony informative sites.

To evaluate if novel clades 1E-I, 1E-II, 1F-I, and 1F-II, uncovered through single marker phylogenetic analysis, represented species of the genus or different taxonomic rank units, we performed pairwise genomic comparisons between all genomes and MAGs, and derived the average amino acid identity (AAI) and the average nucleotide identity (ANIb) indexes (Figure 2; Supplementary Table S4). All of the AAI values obtained from the pairwise comparisons exceeded the recognized thresholds for genus-level differentiation (<65–72%, Konstantinidis and Tiedje, 2007), except for control taxa ‘*F. caldus*’ (average of all igneacidithiobacilli vs. ‘*F. caldus*’ = 69.2%) and *T. tepidarius* (average of all igneacidithiobacilli vs. *T. tepidarius* = 59.1%), which were lower than these threshold values (Figure 2A). These results imply that all genomes and MAGs of novel Fis-tree clades 1E-I, 1E-II, 1F-I, and 1F-II pertain to the ‘*Igneacidithiobacillus*’ genus along with representatives of clades 1B and 1C. In turn, the ANI data (Figure 2B) revealed seven genomic clusters below the 95.9% species threshold, all of which also emerged from the dDDH analysis using the 70% threshold (Supplementary Table S4). Therefore, genomic relatedness indexes support the existence of four novel genomic species within the ‘*Igneacidithiobacillus*’ genus (Supplementary Table S5), which add to the three currently acknowledged species, namely, ‘*Igneacidithiobacillus copahuensis’* (typified by strain VAN18-1^T^), ‘*Ca.* Igneacidithiobacillus yellowstonensis’ (typified by MAG SpSt-908^TS^ as sequence type material), and ‘*Ca.* Igneacidithiobacillus taiwanensis’ single MAG (typified by MAG UBA2468^TS^). Proposed names for these novel species-level rank taxa are: species ‘*Igneacidithiobacillus siniensis’* (typified by strain S30A2^T^), ‘*Ca.* Igneacidithiobacillus chanchocoensis’ (typified by MAG mCHCt20-1^TS^), ‘*Ca.* Igneacidithiobacillus taupoensis’ (typified by MAG TVZ-G3^TS^), and ‘*Ca.* Igneacidithiobacillus waiarikiensis’ (typified by MAG TVZ-G4^TS^). The multiprotein phylogenetic trees shown in Figure 2C; Supplementary Figure S2, which are constructed with a concatenate of 87 universally conserved ribosomal proteins and other phylogenetically informative housekeeping class-core marker genes, and fully supports the differentiation of type strains and type sequence material from the named sulfur-oxidizing species of the *Acidithiobacillia* class and acknowledged species of the genus ‘*Igneacidithiobacillus*’ (Supplementary Table S6).

Basic genomic features of the acknowledged species representatives are shown in Table 2. Data conveyed from strains and MAGs (average estimated completeness 87.9%) show general consistency in terms of size (average 2.4 Mb) and G + C content (average 58.1%), with most species representatives of the genus having fairly small genomes compared with the class average (3.1 Mb, Moya-Beltrán et al., 2021). *I. copahuensis* and *I siniensis*, both species with cultured representatives (average completeness 98.1%), have genome sizes reaching 2.8 Mb, which are comparable to those of its closest genus ‘*Fervidacidithiobacillus’* (average size ‘*F. caldus*’ 2.86 Mb, Valdes et al., 2009; You et al., 2011; Moya-Beltrán et al., 2021). Larger differences and G + C content were observed for MAGs, TVZ-G3, and TVZ-G4.

**Table 2:** Overview of the basic genomic characteristics of the acknowledged ‘*Igneacidithiobacillus*’ spp.

**Species**	**Clade 1C**		**Clade 1B**	**Clade 1E**		**Clade 1F**	
	*‘Igneacidithiobacillus copahuensis’*	**‘*Ca.*** **Igneacidithiobacillus yellowstonensis’**	*‘Igneacidithiobacillus siniensis*’	**‘*Ca.*** **Igneacidithiobacillus taiwanensis’**	**‘*Ca.*** **Igneacidithiobacillus chanchocoensis’**	**‘*Ca.*** **Igneacidithiobacillus taupoensis’**	**‘*Ca.*** **Igneacidithiobacillus waiarikiensis’**
Characteristics	VAN18-1^T^	SpSt908 ^TS^	S30A2^T^	UBA2486^TS^	mCHCt20 ^TS^	TVZ_G3 ^TS^	TVZ_G4 ^TS^
Status	Draft	MAG	Complete	MAG	MAG	MAG	MAG
Completeness (%)	98.15	66.92	98.14	74.24	94.65	95.37	90.28
Contamination (%)	0.00	1.24	0.31	0.03	4.22	0.00	0.62
Strain heterogeneity (%)	0.00	50.00	0.00	0.00	0.00	0.00	0.00
Size (Mbp)	2.77	1.61	2.81	1.69	2.40	1.73	1.83
Coverage (fold)	76.7	5.05	1,472	43	12	14.1	37.1
N50	90,130	3,495	2,782,872	14,238	18,732	41,509	18,296
Contigs (#)	202	497	4	181	306	62	184
GC (%)	58.51	59.03	58.25	59.57	59.20	56.39	55.75
Coding density (%)	93	93	92	90	93	94	94
Genes (#)	2,890	2,113	2,844	2056	2,576	1781	1926
CDS (#)	2,838	2084	2,743	2029	2,522	1734	1887
tRNAs (#)	45	29	48	27	45	41	33
rRNAs (#) 5S/16S/23S	1, 1, 1	-	2, 2, 2	-	2, 1, 1	2, −, −	1, 1, −
Accession	JAAXYO01	DTMS01*	JALQCS01	DDOU01	JAWNZB01	JAEPKX01	JAEPKY01

The results are derived from public draft genomes and MAGs, including a novel MAG reported in the current study.

T stands for type strain.

TS stands for “type by sequence”.

(*) Spst-908 MAG sequence: formerly DTMS01, now SRR7540054.

### Geographical distribution and ecological niche preferences of ‘*Igneacidithiobacillus*’ lineages

**Figure 3:**
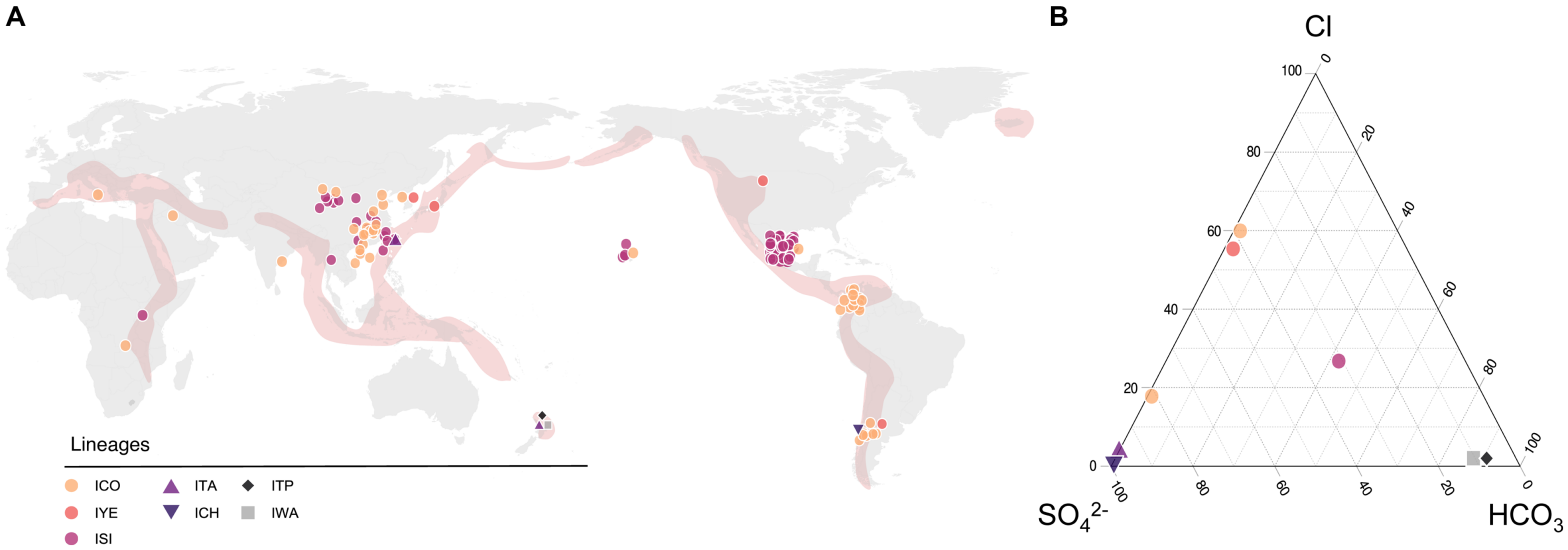
Occurrence of the ‘*Igneacidithiobacillus*’ strains, clones, and MAGs in hydrothermal econiches around the globe. **(A)** Location and relative abundance of each representative according to latitude and longitude coordinates (Supplementary Table S2). **(B)** Ternary plot of the relative concentrations of major anions describing terrestrial hydrothermal systems water chemistry. Concentrations of SO₄^2−^, HCO₃^−^, and Cl^−^ were obtained from the published literature (Hose et al., 2000; Giaveno and María, 2010; Agusto, 2011; Agusto et al., 2012; Rosales Lagarde et al., 2014; Lin et al., 2015; Gaviria Reyes et al., 2016; Muñoz Lopez, 2017; D'Auria et al., 2018; Willis et al., 2019; Zhou et al., 2020b; Sriaporn et al., 2021; Sriaporn, 2022; Barbosa et al., 2023) and this study.

To infer the ecological niches occupied by the recognized *Candidate* species, we evaluated the global occurrence and distribution of members of the ‘*Igneacidithiobacillus’* genus. Environmental and geographical data; associated with each strain, 16S rRNA sequence clone, and/or metagenome-derived genome included in the study, was recovered from the published literature and public databases and used in statistical analysis (Supplementary Table S2). Over 95% of all igneacidithiobacilli strains and sequence clones sampled were mapped to sites along the Ring of Fire in Asiatic countries (China, Taiwan, Korea, and Japan), New Zealand, Chile, Argentina, Colombia, Mexico, and the United States (Figure 3A; Supplementary Table S2), in acknowledged tectonically or geothermally active areas (Renaut and Jones, 2011). Additional origins for ‘*Igneacidithiobacillus*’ spp. could be traced to volcanic sites away from the Ring of Fire such as the Vulcano island in Italy and geothermal areas in central Africa or to sulfide-rich and sulfate-rich waters and soils inland in a number of locations in China, India, and Hawaii. No obvious trend in the geographic distribution of the strains/clones assigned to the different *Candidate* species or phylotypes could be derived from the observed distribution map (Figure 3A) other than the more ubiquitous and cosmopolite occurrence of clade 1B and 1C-I representatives with respect to those of clades 1E and 1F (and probably attributable to sampling biases).

In the analyzed dataset, approximately half of the representative sequences originated from natural environments, such as karst caves, volcanic rivers, sinters, and geothermal pools. The other half of the ‘*Igneacidithiobacillus*’ spp. representatives came from anthropogenic environments (such as mines—heaps, tailings, acid mine drainages, and bioreactors—tanneries, wastewater treatment plants, and sewages) or heavily impacted environments (such as contaminated/polluted water courses or soils). The type of sample from which isolates and clones were recovered included water, sludge, sediment, soil, and snottite. All of these are recognized oligotrophic environments, which are rich in either sulfur, sulfide, or their oxidations products (Parker et al., 2008; Varekamp et al., 2009), and most are also polluted with heavy metals.

#### Clade 1B representatives (*I. sinensis*)

The vast majority of clade 1B members (*n* = 50, originating from a single study; Jones et al., 2016) originate from sulfidic caves Cueva Luna Azufre and Cueva Villa Luz (Tabasco, Mexico) in Los Azufres geothermal field, a hydrothermal spring system in the Mexican Volcanic Axis/Belt (Ferrari et al., 2011). Sequence clones from this set are highly conserved, with less than 0.6% nucleotide sequence variation at the 16S rRNA level. All 50 clones were derived from extremely acidic snottite biofilm samples (pH 0–1.5) dripping from mineral carbonate (limestone) deposits, which were exposed to a hydrogen sulfide-rich atmosphere at cave temperatures ranging from 28 to 30°C (Jones et al., 2016, 2023). One grown representative (AM2) pertaining to clade 1C-I was also recovered from mud samples in this system, having slightly higher temperature (37°C) and pH (pH 1.0–3.0) values (Brito et al., 2014). This implies that different ‘*Igneacidithiobacillus*’ species coexist in the same ecosystem, and however, they partition differentially between habitats (snottites versus sediments). Other clade 1B members (*n* = 23) have been recovered or traced to several mining operations, sharing in common high levels of heavy metal pollution and extremely low pH (Figure 3B; Supplementary Table S2). Although metadata for most of these additional clade 1B representatives are scarce, additional hints on the preferred ecological niche of this group may be derived from a few examples. Clade 1B-uncultured representative K13 originated from total DNA recovered from biopulp samples from a bioleaching reactor that had been inoculated with a mixed bacterial population with a traceable history (Foucher et al., 2003). The inoculum originated from a 10-year-old microbial consortium established from mine waters and sampled by the Bureau de Recherches Géologiques et Minières at a mining site from a gold-bearing arsenopyrite flotation concentrate fed with Kasese cobaltiferous pyrite from Uganda in Africa and stored at 35°C and pH between 1.3 and 1.5. The pH was regulated by adding limestone slurry of 500 g L^−1^ calcium carbonate in the pulp. The presence of limestone in the environment of clade 1B strains seems to be a key factor for their growth, establishment, or prevalence (Figure 3B; Supplementary Table S2), serving either as carbon dioxide source upon limestone dissolution in acid liquors (Esparza et al., 2019; Maltseva et al., 2023) or as a pH buffer (e.g., Sasowsky et al., 1995). Although cultured representatives of the 1B clade (DBS-4, YP-5, 175Fe35, FY-2, TST3, ZJJN-1, ZJJN-2, and ZJJN-3) have not been tested for the effect of calcium carbonate on their growth and/or oxidation performance, they have all been reported to grow in a medium composed of organics (0.01% yeast extract or peptone; Ni et al., 2008; Feng et al., 2012; Li et al., 2018).

#### Clade 1C representatives (*I. copahuensis and ‘Ca.* I. yellowstonensis’)

Clade 1C strains and clones pertaining to subclades 1C-I (a/b sister clades in the 16S rRNA tree) and 1C-II (Figure 1A) come from both natural and anthropogenic environments (Figure 3A; Supplementary Table S2). Subclade 1C-II representatives can be traced to Yellowstone National Park in North America (MAG SpSt908, Zhou et al., 2020a; ‘*Ca.* I. yellowstonensis’, Moya-Beltrán et al., 2021) and the Caviahue-Copahue Volcanic Complex in South America (sequence clone G5, Urbieta et al., 2014). Two cultured representatives clustered in this subclade, ORCS6 and SMK, were recovered from acid sulfate soil samples in Japan (Satoh et al., 2006) and sludge originating in a wastewater treatment plant in Korea (Hassan et al., 2010), respectively. The SpSt908 MAG originates from the metagenome derived from a mud/slurry sample at 66°C and pH 5.0 (OP-RAMG-02, SRA Experiment ID SRX3196666), which was taken from the peripheral area of the Obsidian pool (OP), a persistent thermal spring in the Mud Volcano at Yellowstone National Park (Hamilton-Brehm et al., 2010). In turn, the G5 clone was found in a water pool at Las Maquinas (LMa), a hydrothermal feature from the Caviahue-Copahue Volcanic Complex in Argentina (Urbieta et al., 2014). Both features are shallow thermal pools with mesophilic to thermophilic temperatures (OP: 42–90°C; LMa: 36–93°C) and low to neutral pH (OP: 5.7–6.7; LMa: 2.3–6.7) and contain black sandy material covering the bottom of the pool (Shock et al., 2005; Gaviria Reyes et al., 2016). Both Mud Volcano and Las Maquinas areas are categorized as vapor-dominated with water of distinct sulfated characteristic generated by the condensation of ascending vapor from the geothermal reservoirs (e.g., sulfide or sulfur dioxide) in the groundwater (Kennedy et al., 1985; Fournier, 1989; Varekamp et al., 2009; Agusto, 2011; Gaviria Reyes et al., 2016). As they surface, sulfide is oxidized resulting in acidic fluids of elevated sulfate concentrations that drive chemical weathering of the rock beds, leading to elevated concentrations in solution.

Subclade 1C-Ib entails strains from a gold-bearing mine site (El Zancudo gold mine, Titiribi, Antioquia, Colombia). El Zancudo gold mine abandoned drainage, resulting from sulfide mineral weathering (pyrite, arsenopyrite, and galena), has near neutral pH 6.5–7.4 due to the buffering capacity of several carbonate minerals (dolomite, aragonite) present at the site (Barragán et al., 2020). Interestingly, all these strains (IBUN_Pt1247) were isolated from enrichments with arsenate and showed high resistance to the metalloid. In turn, subclade 1C-Ia grouped a more diverse set of strains and sequence clones, originating from acidic pools and rivers sourced by volcanic fluids and/or gases, heavily polluted water courses (such as those associated with mine drainages or tanneries), or soils. Sampling sites had in common low pH, moderate to high temperature, and high osmotic strength, which was conveyed either by sulfate ions or chloride salts, and high to extremely-high concentrations of different sorts of heavy metals (Figure 3B; Table 1; Supplementary Table S2).

#### Clade 1E-1F representatives (*‘Ca.* I. taiwanensis’, *‘Ca.* I. chanchocoensis’, *‘Ca.* I. taupoensis’, and *‘Ca.* I. waiarikiensis’)

Subclade 1E representatives remain uncultured and were typified based on metagenomic sequence bins, which were recovered from geothermal pools or their outflows. Two of them group together in subclade 1E-I; UBA2486, a MAG recovered from the Shi-Huang-Ping thermal manifestation at the Tatun Volcanic Group in Taiwan (Lin et al., 2015) and TVZ_G2, a MAG recovered from the Tikitere springs at the Taupo Volcanic Zone (TVZ) in New Zealand (Sriaporn et al., 2021). A third MAG (mCHCt20-1) of this clade (subclade 1E-II) was recovered from a thermal feature in the Caviahue-Copahue Volcanic Complex, specifically from ChanchoCó located on the Chilean side of the volcano. The samples from which the three MAGs were derived share mildly acidic pH values (~ pH 3) and moderate to high temperatures (> 40°C).

Subclade 1F is also defined by two MAGs deriving from sulfur-rich hot springs at Tikitere in the TVZ area (Sriaporn et al., 2021). The MAG TVZ_G3 (subclade 1F-I) was obtained from metagenomes recovered from protrusive stromatolitic siliceous deposits (digitate sinter) at the air–water interface in the hot spring margins and outflow channels at a location designated as TIK2 (Sriaporn et al., 2020). In turn, MAG TVZ_G4 (subclade 1F-II) was obtained from subaqueous sediments (a few mm below the water surface) from the same hot spring. This is the same site from which ‘*Ca.* I. taiwanensis’-related MAG TVZ_G2 was obtained, implying that different ‘*Igneacidithiobacillus*’ spp. coexist in the same hot spring, albeit partitioning differentially between subaerial and subaqueous microhabitats (Figure 3B; Table 1; Supplementary Table S2). Both are influenced by the chemistry of the geothermal waters of the Cooking Pool hot spring, which are classified as acid-sulfate-bicarbonate (ASB) waters (Sriaporn et al., 2020), and its characteristics are attributed to underground gases, reacting with local rocks. At sampling, the TIK2 hot spring had a water temperature ranging between 39.7 and 48.5°C and a pH of 5.9 (Sriaporn et al., 2020).

This data suggest the igneacidithiobacilli have a wider pH range and endure lower pH and higher temperatures than other *Acidithiobacillia* class members. In addition, they partition differentially between aerial, interfacial, and solids in their preferred habitats, according to gradients in oxygen and other gases serving as electron donors or acceptors, and have in common increased resistance to high ionic strength liquors and toxic metals.

### Pangenome analysis reveals minimal core functions of the igneacidithiobacilli

To obtain a comprehensive view of the distinctive phenotypic properties of the group, we next compared the available genomes and MAGs of ‘*Igneacidithiobacillus*’ spp. (*n* = 14, Table 2) and reconstructed relevant aspects of their metabolism. To this end, proteins were grouped by orthology into protein families, and their occurrence and conservation were evaluated against the class-wide pangenome. The igneacidithiobacilli (encompassing 14 genomes of 7 species) harbored 5,415 distinct PFs, which was significantly lower than the pangenome size of the monospecific genus *‘Fervidacidithiobacillus*’ comprising 7,127 PFs across 18 genomes (Moya-Beltrán et al., 2021). While the reduced number of PFs could be attributed to the inclusion of several MAGs potentially lacking some genes, genomic completeness deduced from the occurrence of 258 universal protein markers (as implemented in CheckM, Parks et al., 2015) averaged 98.15% for genomes and varied from 66.92 to 95.37% for MAGs, with five of the seven MAGs meeting the established high-quality threshold (> 90% completeness, Bowers et al., 2018). In addition, the core/pangenome ratio of these two genera was comparable (‘*Igneacidithiobacillus*’: 16.5%; *‘Fervidacidithiobacillus*’: 17.2%; Moya-Beltrán et al., 2021), implying that the accessory gene complement of the igneacidithiobacilli is currently under-sampled.

**Figure 4:**
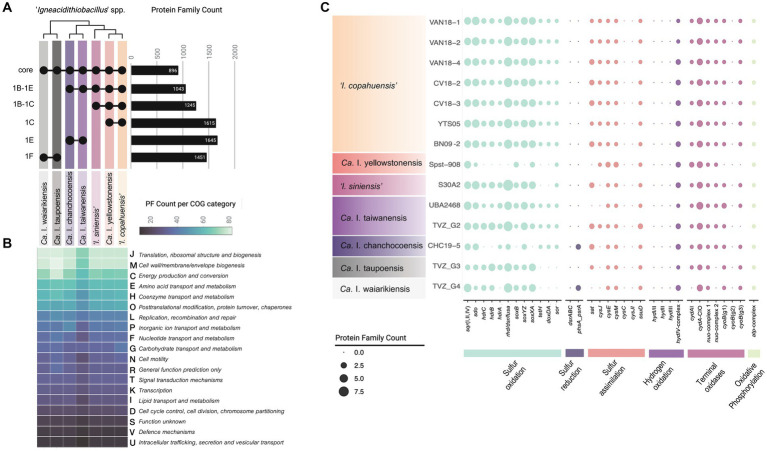
Occurrence and abundance of protein families encoding traits common to all ‘*Igneacidithiobacillus*’ spp. **(A)** Upset plot of the distribution of PFs shared between igneacidithiobacilli clades and species derived from pangenome analysis (core PFs). Clades are those derived from the core proteins phylogenetic tree shown in Figure 2C. **(B)** Number of genus-level core PFs associated with general COG functional categories per lineage ordered from most frequent to less frequent categories in the set. **(C)** Phyletic patterns of relevant energy metabolism genes (and associated gene clusters). Color coding is as labeled in the figure panel.

A total of 896 PFs were shared (core) among representatives of the seven ‘*Igneacidithiobacillus*’ lineages (Figure 4A). This core represents 31.6% of the predicted proteome of `*I. copahuensis*´ VAN18-1 type strain (2,838 CDSs; 2,749 PFs), being slightly higher than the percentage of core proteins carried by other *Acidithiobacillia* (e.g., 21.4% in *Acidithiobacillus* spp.; Moya-Beltrán et al., 2021). The number of shared genes rose in correlation with increasing genetic relatedness among the species, amounting to 1,043 for clade 1B-1E, 1,245 for clade 1B-1C members, and 1,615 for `*I. copahuensis*´ strains (clade 1C-I), the species of the genus with the largest number of cultured and sequenced representatives (Figure 4A). These numbers reflect relevant gene gains and losses that occurred during the differentiation of these lineages. Core genes partitioned into 21 COG categories and 25 KEGG paths and included most housekeeping genes required for basal metabolism of *Acidithiobacillia* class members (Moya-Beltrán et al., 2021), encompassing essential information processing genes, chemolithoautotrophic energy metabolism, and cell envelope biogenesis and maintenance genes (Figure 4B; Supplementary Table S7).

Sulfur metabolism enzyme-encoding genes required for the utilization of hydrogen sulfide (*sqr*), elemental sulfur (*sdo, sor*), and thiosulfate (*sox*) as electron donors, as well as those encoding key respiratory chain elements (*cydA-CIO, cyoB, nuo-complex, and atp-complex*), were present and conserved with respect to known acidithiobacilli (Figure 4C), strongly suggesting that the igneacidithiobacilli are also obligate chemolithotrophs. Some of these genes or complexes varied in copy number, with enrichment of functions in particular lineages (e.g., 4–5 copies of *sdo* in ‘*I. copahuensis’*) and depletion of functions in others (e.g., 1 copy of the *nuo* complex in ‘*Ca.* I. taupoensis’ and ‘*Ca.* I. waiarikiensis’).

As other *Acidithiobacillia* class members, ‘*Igneacidithiobacillus*’ seem to be incapable of dissimilatory sulfate reduction, lacking key functional markers for this process (*dsrABC*, Thauer et al., 2007). They also lack the *sreABCD* genes that enable anaerobic growth on sulfur coupled to ferric iron reduction in iron/sulfur-oxidizing *Acidithiobacillus* spp. (e.g., Osorio et al., 2013). Yet the MAGs of lineages ‘*Ca.* I. chanchocoensis’ and ‘*Ca.* I. waiarikiensis’, encode a thiosulfate/polysulfide reductase (*phs/psr*) known to catalyze the stepwise reduction of elemental sulfur and the zero-valent sulfur of thiosulfate or polysulfides to sulfide in the absence of oxygen (Heinzinger et al., 1995; Jormakka et al., 2008).

Certain igneacidithiobacilli (‘*Ca.* I. yellowstonensis’, ‘*I. siniensis*’, ‘*Ca.* I. taupoensis’, and ‘*Ca.* I. waiarikiensis’) seem also unable to derive energy from tetrathionate, lacking the genes encoding not only the tetrathionate hydrolase (*tetH*) but also the membrane-bound tetrathionate forming thiosulfate:quinone oxidoreductase (*doxDA*). Lineages missing these PFs are the ones originating from the highest temperature habitats, where tetrathionate would be particularly unstable in the presence of strong reductants such as sulfide (Xu et al., 1998), and which is highly abundant in most of these habitats. Genes for the assimilation of sulfur into the cysteine biosynthetic pathway identified in the ‘*Igneacidithiobacilllus*’ spp. included those involved in the acquisition of sulfur from sulfate (*sat, cysJ, cysE, and cysM*) as other *Acidithiobacillia* class species (Valdés et al., 2003), and from sulfonates (*ssuD*); so far, the capacity has been overlooked in this group of acidophiles (Supplementary Table S7). Lack of the APS kinase (*cysC*) and the sulfite reductase (*cysJI*) suggests that thiosulfate—instead of sulfate—may be used as sulfide donor in the synthesis of cysteine by CysM (Zhao et al., 2006).

Several core PFs (*n* = 29) occurred in gene dosages are higher than 2, including relevant functions for carbohydrate transport and metabolism (*n* = 8) involved in glycolysis (*pdhB, pdhC, pgi, and gpmI*), pyruvate metabolism (*ackA*), pentose phosphate metabolism (*zwf*), carbon fixation through the Calvin-Benson-Bassham reductive pentose phosphate cycle (*rbcL*), glycogen utilization (*glgP, cga*), and energy metabolism (*cydAB*), among others (Supplementary Table S7, core PFs). Several of these genes provide the igneacidithiobacilli with the capacity to fix carbon dioxide autotrophically.

Exclusive gene complements (present in all genomes/MAGs) of each lineage were predominantly hypothetical functions and variants of PFs, existing in other lineages, providing little insight into the adaptive features, and differentiating these species. Lineage-specific PFs with predicted functional assignments included genes involved in: (a) defense against foreign DNA which were particularly overrepresented in ‘*Ca.* I. yellowstonensis’, ‘*I. siniensis*’, and ‘*Ca.* I. chanchocoensis’, (b) osmotolerance preservation which potentiated different physiological strategies in each species, (c) cell envelope integrity and modification which entailed a wide diversity of species-specific glycosyltransferases, and (d) both uptake and efflux transporters of different sorts in each lineage.

### Recorded and inferred morphophysiological traits distinguish *candidate* species

To ascertain the physiological characteristics of the novel ‘*Igneacidithiobacillus*’ spp., we analyzed common and differential genome-derived traits in the light of emerging trends in the published metadata for the reassigned strains and clones (Supplementary Figure S3). Preferential temperature, pH, and water chemistry of the selected representatives of the igneacidithiobacilli lineages derived from available reports in the literature (and/or generated herein) on the source habitat, isolation conditions, or growth experiments are shown in Table 3. Clade 1C representatives (‘*I. copahuensis’*, ‘*Ca.* I. yellowstonensis’) have been categorized as thermotolerant (Satoh et al., 2006; Hassan et al., 2010; Li et al., 2018; Moya-Beltrán et al., 2021), while clade 1B representatives (‘*I. siniensis*’) have been reported to endure extremely low pHs (Ni et al., 2008; Feng et al., 2012; Jones et al., 2016; Liang et al., 2019). In turn, clade 1E lineages originate from mildly acidic habitats with higher temperature maxima (Lin et al., 2015), and clade 1F representatives originate from mildly thermal environments of higher pH (Sriaporn et al., 2020, 2021). The habitats of origin differ also in their reported water chemistry, having relevant differences in the contents of chloride salts, carbonates, and metals. We thus assessed the distribution of genes encoding products that could confer the igneacidithiobacilli lineages with differential growth capacities based on these habitat and growth characteristics.

**Table 3:** Physicochemical conditions and characteristics of samples/sites of origin of ‘*Igneacidithiobacillus*’ spp.

**Clade**	**#/clade** ^**a**^	**Representative**	**T (°C) habitat** ^**b**^	**T (°C) growth or isolation** ^**c**^	**pH habitat** ^**b**^	**pH growth or isolation** ^**c**^	**Water chemistry** ^**d**^
1B	73	S30A2	26–30	28–40	0.0–3.4	1.5–5.0	AS, ASB
							SO_4_^2−^ 960 mg/L
							Cl^−^ 782 mg/L
							HCO_3_^−^ 1,310 mg/L
1C_II	1	SpSt908	42–90 (66)		5.0–6.7	–	AS
							SO_4_^2−^ 231 mg/L
							Cl^−^ 278 mg/L
1C_Ia	3	LMa36_G5	36–93	–	2.3–6.7	–	AS
							SO_4_^2−^ 4,055 mg/L
1C_Ib	13	IBUN_ Pt1247_S3	21.5	28–32	5.6–7.4	1.5–3.5	ASB
							SO_4_^2−^ ND
							HCO_3_^−^ ND
1C_Ic	34	VAN18-1	37–40	28–40	2.2–2.5	2.5–2.8	AS, ASC (*)
							SO_4_^2−^ 4,495-20590 mg/L
							Cl^−^ 437-8283 mg/L
1E_I	2	UBA_2486	50–85	–	2.7–3.25	–	AS
							SO_4_^2−^ 378 mg/L
1E_II	1	mCHCt20-1	38.9–56	–	5.8–7.0	–	AS
							SO_4_^2−^ 2,165 mg/L
1F_I	1	TVZ_G3	38.9	–	5.9	–	ASB
							SO_4_^2−^ 33 mg/L
							HCO_3_^−^ 359 mg/L
1F_II	1	TVZ_G4	38.9	–	5.9	–	ASB
							SO_4_^2−^ 33 mg/L
							HCO_3_^−^ 359 mg/L

Acid-sulfate (AS), acid-sulfate-bicarbonate (ASB), acid-sulfate-chloride (ASC), no data (ND).

^a^Total number of strains, sequence clones, and/or MAGs assigned to the clade.

^b^Physicochemical values derived for the habitat occupied by strains, sequence clones, and/or MAGs analyzed in this study.

^c^Physicochemical values of growth derived for strains during isolation (enrichment) and/or growth in pure culture.

^d^The water chemistry of the habitats of each clade, recovered from available literature (references stated in Figure 3, additional information can be found in Supplementary Table S2) or generated in this study (*). The concentration of sulfates was determined by absorbance at 420 nm as by Kolmert et al. (2000) and the concentration of chloride by mercuric nitrate titration (Roberts et al. 2003).

#### Thermal and low pH adaptation traits and genes

Known features that confer adaptation to both high temperature and low pH include the synthesis of more saturated and longer chain fatty acids (e.g., straight-chain saturated fatty acids, Hazel and Williams, 1990; Mykytczuk et al., 2010) and triterpenoids (hopanoids, Driessen et al., 1996), which increase membrane rigidity and stability, enhanced mechanisms for DNA repair to counteract the increased DNA damage caused by stress (Feng et al., 2019), and increased protein stability and turnover (Li et al., 2005). All ‘*Igneacidithiobacillus*’ spp. analyzed encode genes for the biosynthesis of saturated fatty acids (*accABCD, fabDHGZIF*) while lacking the genes (*fabAB-fadR*) required for the synthesis of unsaturated fatty acids (Supplementary Table S7), which disrupt the order of the phospholipid bilayer (Zhang and Rock, 2008). These results suggest that membranes of the igneacidithiobacilli are enriched in efficiently packed saturated fatty acids with low permeability properties. In addition, ‘*Igneacidithiobacillus*’ spp. genomes/MAGs were found to encode genes for the biosynthesis of hopanoids (*hpnABFGHIJKLMN*) (Supplementary Table S7), as all acidophilic members of the *Acidithiobacillia* class do (González-Rosales et al., 2022), which have been shown to decrease membrane fluidity in bacteria under pH stress (Sáenz et al., 2015).

All ‘*Igneacidithiobacillus*’ spp. encode a vast repertoire of DNA repair involved in base excision repair (BER, *n* = 10), nucleotide excision repair (NER, *n* = 5), mismatch repair (MMR, *n* = 15), and recombinational repair (RR, *n* = 17) (Supplementary Table S7). Most of these are enzymes conserved universally across bacteria and *Acidithiobacillia* class members (Cárdenas et al., 2012), while other DNA repair enzyme-encoding genes are present in high gene doses, in particular species of ‘*Igneacidithiobacillus*’ (e.g., the DNA MMR protein MutS/S2 with a minimum of 4 copies/genome in ‘*Ca.* I. taiwanensis’ and a maximum of 7 copies/genome in *I. copahuensis*), or are clearly differential, e.g., the nucleotide pool-sanitizing enzyme MutT (Ito et al., 2005) is absent in ‘*I. copahuensis’*. Other examples of these differentially distributed capacities are shown in Supplementary Table S7.

In addition, most of the genes that make up the proteostasis network in acidophiles, including highly redundant genes in *Acidithiobacillia* spp. that encode for the periplasmic chaperone HtrA and the proteolytic ATPase Lon (Izquierdo-Fiallo et al., 2023), are found in the igneacidithiobacilli genomes/MAGs analyzed herein. Enrichment in thiol–disulfide interchange protein DsbG responsible for the formation and rearrangement of disulfide bonds during the folding of secreted and membrane proteins in bacteria and for protecting free cysteines in proteins from sulfenylation (Kadokura and Beckwith, 2010) was found in ‘*I. copahuensis’* and ‘*I. siniensis*’ with 7–10 copies/genome of *dsbG* compared with other spp. (2–4 copies/genome). In addition to this, all ‘*Igneacidithiobacillus*’ spp. encode an extensive repertoire of chaperones, proteases, and peptidases, which are predicted to contribute to protein stabilization and turnover in this taxon (Supplementary Table S7).

Comparison of *Acidithiobacillia* class core proteins provides additional insights into the adaptation of ‘*Igneacidithiobacillus*’ lineages to thermal environments (Supplementary Figure S4), with species occupying the highest temperature habitats having increased proline content (which enhances thermal stability) and decreased glycine and alanine content (which enhances protein flexibility), as well as decreased abundance of amino acids that form hydrogen bonds (such as threonine) or that are prone to deamidation at high temperatures and low pH, such as asparagine and cysteine (Hait et al., 2020; Ahmed et al., 2022).

Most genes known to occur on the *Acidithiobacillia* class representatives and other acidophiles that are deemed necessary for acid tolerance (Baker-Austin and Dopson, 2007; González-Rosales et al., 2022), namely in charge of restricting proton entry by lowering membrane permeability (see above), reversing the membrane potential of the cytoplasmic membrane taking up positively charged potassium ions, purging of protons using diverse sorts of H^+^ efflux and antiport mechanisms, and consuming protons via decarboxylation and oxidative phosphorylation reactions, were found to occur in igneacidithiobacilli lineages albeit with some qualitative and quantitative differences (Supplementary Table S7). Variations in gene dose for these PFs always favored 1B/C clade representatives, which may explain the observed differences in the occupancy of habitat or microhabitats.

#### High osmolarity adaptation traits and genes

Strains in clade 1C have been shown to grow and oxidize elemental sulfur or tetrathionate in the presence of NaCl (e.g., strain V1, Norris et al., 2020). Strain V1, isolated from Vulcano, Italy, showed salt tolerance with an upper limit of 0.85 M NaCl, and after preadaptation to NaCl, it could withstand 1.5 M of this salt. It also endured high osmotic pressure, resulting from similar molarities of sodium ions from sulfate salts. Published studies report other 1C clade strains as sulfate-tolerant bacteria (AZ11, Lee et al., 2003; Tt, Kumar et al., 2008), e.g., AZ11, showing sulfur-oxidizing activity in the presence of 80 g/L sulfate accumulated in the growth medium (Lee et al., 2003). High sulfate concentrations have been traced to several of the sites of origin of 1C clade strains (Supplementary Table S2).

**Figure 5:**
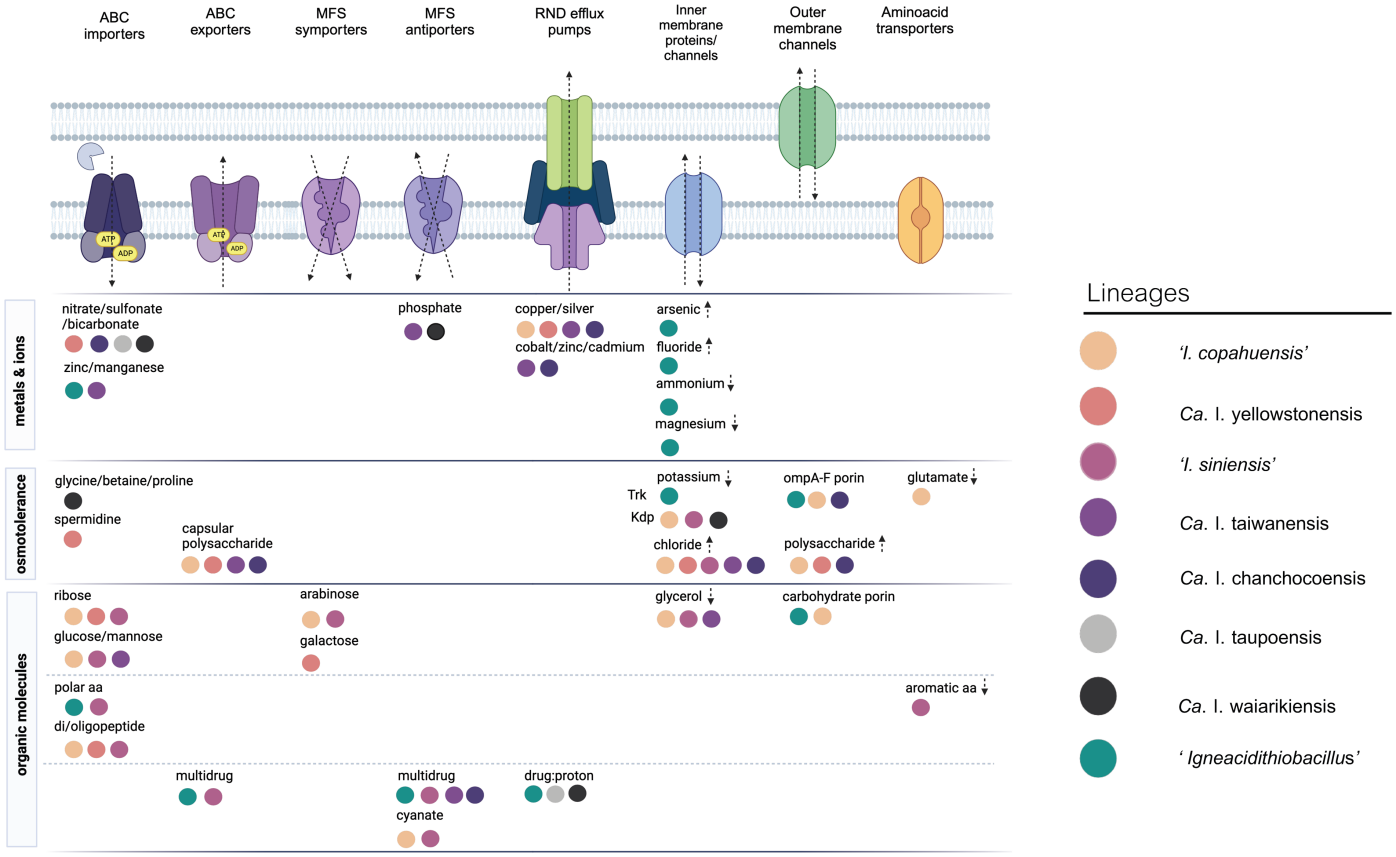
Transporters found in the exclusive gene complement of the different ‘*Igneacidithiobacillus*’ spp. Data depicted in the figure represents a selection from the full length list of transporters shown in Supplementary Table S7. Created with BioRender.

Genome-based metabolic reconstruction analysis revealed the presence and conservation of the canonical mechanisms described in other acidophiles to cope with the osmotic stress (Rivera-Araya et al., 2019). All members of the genus encode K^+^ transporters to meet the cellular demand for potassium and keep the cellular turgor in response to osmotic upshift (Supplementary Table S7), including the low-affinity TrkA potassium uptake protein (*n* = 2) and the high-affinity potassium uptake system YggTS (*n* = 1), while ‘*I. copahuensis*’, ‘*I. siniensis*’, and ‘*Ca.* I. taupoensis’ also encode the high-affinity potassium multi-subunit uptake transporter complex KdpABCDE (*n* = 1) (Figure 5). Evidence for the presence of mechanisms to let out the K^+^ surplus was also found in each genome/MAG, including the MscK/KefA potassium efflux system (*n* = 2–3) and several mechanosensitive channels (MscL *n* = 1, MscS/YggB *n* = 1–4) which are predicted to help maintain the intracellular K^+^ concentrations regulated and fine-tune cell turgor in these taxa, as has been shown to occur in other bacteria (Kung et al., 2010). In addition, several bacterial chloride channel (CLC) homologs that were predicted to mediate the exchange (antiporting) of chloride ions and protons through the membrane in response to concentration gradients of either chloride ions or protons (Matulef and Maduke, 2007) were identified in the ‘*Igneacidithiobacillus*’ genomes/MAGs, except for ‘*Ca.* I. taupoensis’ and ‘*Ca.* I. waiarikiensis’ (Figure 5; Supplementary Table S7). These variations in gene dosage could have a functional significance in osmotolerance of the different lineages of the genus.

All ‘*Igneacidithiobacillus*’ lineages were predicted to be able to produce and/or uptake osmoprotectans. Among the amino acids (1)commonly used as osmoprotectants, these lineages can synthetize glutamate (glutamate synthase GOGAT, *gltBD*; ATP-dependent glutamine synthetase GS, *glnA*) which acts major counterion to counterbalance the high levels of potassium accumulated during osmotic stress in different bacteria (e.g., Goude et al., 2004), and (2) its decarboxylation product gamma-aminobutyric acid [glutamate decarboxylase (EC:4.1.1.15), *gadB, gadA*, GAD], (3) proline from glutamate and ATP [Glutamate 5-kinase (EC 2.7.2.11) ProB, Gamma-glutamyl phosphate reductase (EC 1.2.1.41) ProA, Pyrroline-5-carboxylate reductase (EC 1.5.1.2) ProC] and (4) glycine from carbon dioxide and ammonia through the Glycine Cleavage System working in reverse (GcvHPBPATR), or through the glycine hydroxymethyltransferase pathway [(EC:2.1.2.1) K00600, GlyA].

No genes for the biosynthesis of hydroxy/ectoine, glycine-betaine, sarcosine, or taurine could be found in the genomes analyzed; yet, genes encoding the proline uptake transport system ProXWV were found in ‘*Ca.* I. waiarikiensis’, suggesting that proline and alternative compatible solutes for which this transporter has affinity (glycine betaine, proline-betaine, carnitine, and ectoine) may be taken up from environmental sources and reduce the energy spent on salt and/or drought adaptation (Imhoff et al., 2020). All igneacidithiobacilli encode the genes required for the biosynthesis of both putrescine and spermidine, including arginine decarboxylase (*speA*), agmatine deaminase (*aguA*), and N-carbamoylputrescine amidohydrolase (*aguB*). These enzymes decarboxylate arginine to agmatine and, subsequently, convert it into putrescine. The genes encoding the enzymes that catalyze the conversion of putrescine to spermidine were also identified in the genomes/MAGs analyzed, including *speE* and *speD* (Supplementary Table S7). These genes encode the spermidine synthase SpeE and the SAM decarboxylase SpeD (Shah and Swiatlo, 2008).

In addition, the ability to synthesize glycerol (from phosphatidylglycerol, cardiolipin synthase A/B [EC:2.7.8.-], K06131, ClsA_B and from D-glycerate, alcohol dehydrogenase (NADP+) [EC:1.1.1.2], YahK) and break it down (to dihydroxyacetone phosphate, phosphoenolpyruvate: dihydroxyacetone phosphotransferase [EC:2.7.1.121] K05878/9, DhaKL) was found in all ‘*Igneacidithiobacillus*’ analyzed (Supplementary Table S7). However, no evidence for the capacity to use trehalose, sorbitol, or mannitol as osmoprotectants could be inferred from the genomes/MAGs. Of all known compatible solutes, glycerol is the simplest and cheapest to produce, being generally found in organisms that grow at the highest salt concentrations (Oren, 1999), suggesting that all members of the genus are equally suited to mount this response.

#### Traits and genes required for the adaptation to organic compounds and metals

Growth of some clade 1B strains has been shown to be mildly promoted in sulfur medium supplemented with 0.01% yeast extract and/or peptone, rather than in their absence (Ni et al., 2008; Feng et al., 2012), suggesting that these strains are favored by an organic source of nitrogen or are limited by certain amino acids and/or vitamins or that their environment may have a shortage of nitrogen. Consistently, all the ‘*Igneacidithiobacillus*’ genomes/MAGs analyzed encoded 1–2 copies of the polar amino acids (serine, threonine, asparagine, glutamine) uptake protein (K02030), and several of them also harbor within their species-exclusive gene repertoires several genes encoding amino acid transporters (n = 13), such as the general aromatic amino acid permease AroP found in the clade 1B sequenced representative, the serine permease YjeM in ‘*Ca.* I taupoensis’, or less specific di/oligopeptide ABC transporters (Figure 5; Supplementary Table S7). Comparative metabolic reconstruction of the ‘*Igneacidithiobacillus*’ genomes/MAGs showed that they all lack the genes encoding the enzyme D-3-phosphoglycerate dehydrogenase (EC 1.1.1.95), which catalyzes the first committed and rate-limiting step in the phosphoserine pathway of serine biosynthesis; asparagine synthetase [glutamine-hydrolyzing] (EC 6.3.5.4), histidinol-phosphatase (EC 3.1.3.15), and phosphoribosyl-AMP cyclohydrolase (EC 3.5.4.19) required for histidine biosynthesis, suggesting that the members of the genus may all be serine, asparagine, and histidine auxotrophs (Supplementary Table S7).

In common, both 1C (e.g., strains Tt and SMK, clone Fe812) and 1B clade members (e.g., strains 175FE35 and NJU-AMD3, clones X2, S24) have been reported to have high tolerance to heavy metals, being isolated from waters and sediments, rich in metals and metalloids, and/or from heavily polluted soils (Kumar et al., 2008; Zhu et al., 2014). In several cases, strains of these clades have been enriched or selected upon stress caused by heavy metals or incremental exposure to heavy metals and metalloids, such as arsenic (e.g., Barragán et al., 2021), cadmium (e.g., clone Fe812, Tan et al., 2016), and chromium (e.g., Zeng et al., 2016), among others (Kumar et al., 2008; Zhu et al., 2014). All ‘*Igneacidithiobacillus*’ genomes/MAGs are endowed with genes encoding the thioredoxin-dependent arsenate reductase [(EC:1.20.4.4), *arsRBC-arsH*], and many of them also with the mercuric reductase [(EC:1.16.1.1), *merA*]. At least one cation diffusion facilitator (CDF) for Co/Zn/Cd similar to ZitB (Grass et al., 2001; Wang et al., 2012) and CzcD (Alquethamy et al., 2020) that mediates zinc export in Gram-negative bacteria via an antiport mechanism (Guffanti et al., 2002) and one P-type copper exporting ATPase similar to CopA (Rensing et al., 2000), were found in all igneacidithiobacilli genomes analyzed. Several resistance-nodulation-cell division (RND) superfamily efflux transporters of undefined specificity, yet similar to known systems used in the extrusion of heavy metals such as Co/Zn/Cd via the CzcCBA family (Blencowe and Morby, 2003) or Cu/Ag via the CusCFBA family (Conroy et al., 2010), were also identified in the different lineages, being two-fold more abundant in the clade B members (‘*I. copahuensis*’, ‘*Ca.* I. yellowstonensis’, ‘*I. siniensis*’) than in the other species representatives analyzed (Figure 5; Supplementary Table S7). An additional transporter, predicted to have specificity for chromate based on its similarity to the plasmid-encoded chromate transporter ChrA proteins from *Alcaligenes eutrophus* [ChrA(Aeu)] (Nies et al., 1990) and *Pseudomonas aeruginosa* [ChrA(Pae)] (Cervantes et al., 1990), was also found in ‘*I. siniensis*’, suggesting that this strain may be uniquely chromate-resistant.

#### Observed and predicted morphological traits

Morphologically, strains of this clade resemble other species of the *Acidithiobacillia* class, being short rods endowed with flagella (Feng et al., 2012) or motile (Li et al., 2018). A tuft of flagella has been described based on transmission electron microscopy (TEM) imaging in ZJJN strains (1B) (Feng et al., 2012); yet, images are not very clear. ‘*I. copahuensis’* strains, represented by VAN18-1, are highly motile when grown in acidified mineral salt medium (pH 2.5) at 40°C, containing trace elements (Dopson and Lindstrom, 1999) and 5 mM tetrathionate as energy sources (Supplementary Multimedia File S1). Genome-based analysis of the identified ‘*Igneacidithiobacillus*’ species confirms this trait as widespread in genus (Supplementary Table S7), with predicted flagellar gene products having amino acid sequence identity of >60%.

**Figure 6:**
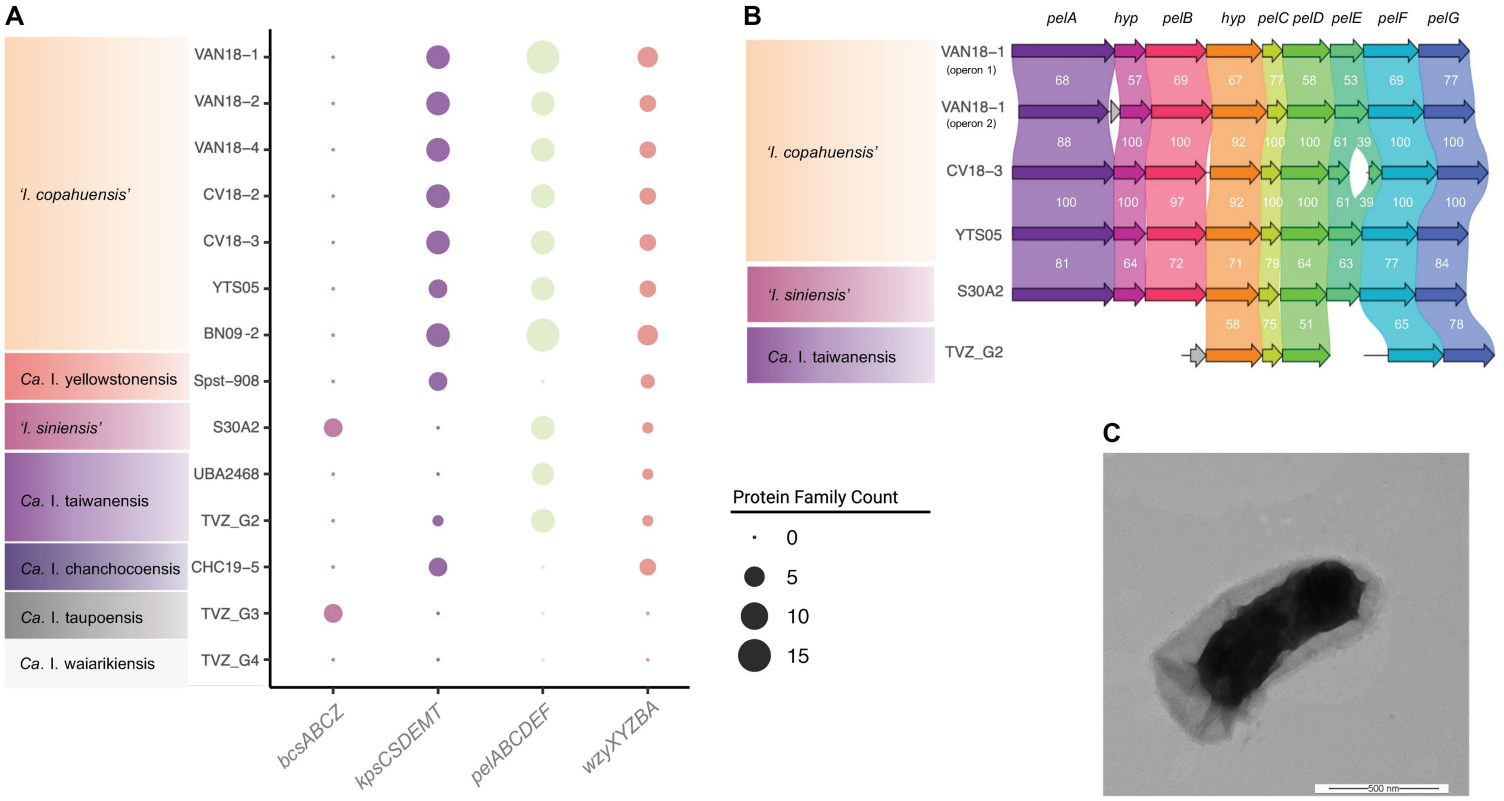
Morphophysiological characteristics of ‘*Igneacidithiobacillus*’ spp. **(A)** Phyletic patterns of relevant genes involved in capsule and extracellular matrix polysaccharide biosynthesis and/or transport (and associated gene clusters). The size of the dots represents the protein family count scored per cluster type. **(B)** Pel polysaccharide gene cluster organization and conservation across igneacidithiobacilli. ‘*I. copahuensis’* strain VAN18-1 encodes 2 gene clusters, noted as *pel-1* and *pel-2*. The *pel-2* gene cluster is present in other strains of the species and in ‘*I. siniensis*’ and ‘*Ca. I. taiwanensis*’. Numbers correspond to the average percent similarity between reciprocal protein pairs as inferred with clinker (Gilchrist and Chooi, 2021). **(C)** Transmission electron micrograph (4.3 K X) of strain ‘*I. copahuensis’* VAN18-1 grown in mineral salt medium containing trace elements (Dopson and Lindstrom, 1999) in the presence of 5 mM tetrathionate at pH 2.5 and 40°C. Bar: 500 nm.

All analyzed igneacidithiobacilli encode the genes (*rmlABCD*) required for the biosynthesis of L-rhamnose containing polysaccharides (such as lipopolysaccharides and extracellular and/or capsular polysaccharides; Li et al., 2022), and their secretion to the bacterium–environment interface. ‘*I. copahuensis*’ genomes also encode ABC transporter-dependent (*kpsCSDEMT*) and the synthase-dependent (*pelABCDEFG*) extracellular polysaccharide synthesis machinery (Figure 6A), which is involved in the biosynthesis of capsule and pellicle biofilms, respectively (Whitfield et al., 2020). The presence of several of the *pel* gene-cluster genes suggests that ‘*I. siniensis*’ and ‘*Ca.* I. taiwanensis’ also share the capacity to produce extracellular matrix Pel polysaccharide (Figure 6B). In the rest of the igneacidithiobacilli, these gene clusters are either incomplete or completely lacking, e.g., in clade 1F. In addition, both 1B and 1C clade representative strains have been observed to have thick capsules around the cells in TEM micrographs (Feng et al., 2012; Figure 6C), which possibly contribute to species survival and adaptation to the harsh (and fluctuating) conditions of their environment.

Variations in the repertoire of species-exclusive glycosyltransferases interspersed in the gene clusters encoding the molecular machinery for the biosynthesis of capsule the O-antigen polysaccharide of lipopolysaccharides (LPS), and other envelope-related structures, were also observed (average of seven/genome-MAG), suggesting that ‘*Igneacidithiobacillus*’ spp. have distinct cell surface modifications (Supplementary Table S7), many of which may confer each lineage with specific adaptive advantages (e.g., to different abiotic stress conditions).

## Conclusion

In this study, we combined a comprehensive phylogenetic analysis of publicly available 16S rRNA gene sequences from un/mis/assigned strains and uncultured sequence clones sampled globally, and genomic taxonomy methods applied to genomes and MAGs of selected representatives of the *Acidithiobacillia* class, to expand the contours of the genus ‘*Igneacidithiobacillus*’. Multiprotein phylogenomic trees supported the existence of at least seven species, four of which remained uncharted before this study. Members of the genus shared strong ecological preferences for tectonically active geothermal regions, predominantly along the Ring of Fire, occurring also in oligotrophic environments enriched in sulfur compounds. Metadata analysis revealed distinct habitat preferences among the specific lineages with respect to temperature, pH, and water chemistry, providing grounds for future prospective sampling and isolation of culturable representatives of the taxon. Compared with most acidophiles (including the majority of members of the *Acidithiobacillia* class), ‘*Igneacidithiobacillus*’ spp. appear to endure a wider range of pH and a lower pH limit along with higher temperatures (likely also, variations in these parameters) and higher ionic strengths. Genomic comparisons revealed that the gene repertoire of igneacidithiobacilli, and particularly the accessory gene complement, is still under sampled. Despite this fact, lineage-specific variations in occurrence and/or dosage of protein families related to high temperature and low pH adaptation, DNA repair mechanisms, tolerance to osmotic stress and heavy metals, and others responsible for cell surface modifications shed light on the adaptive traits of these lineages. Altogether, the findings presented herein provide insights into the genomic landscape of the novel genus ‘*Igneacidithiobacillus*’ and its diversity, laying the groundwork for further research on their ecology, evolution, and biotechnological potential.

## Data availability statement

The datasets presented in this study can be found in online repositories. The names of the repositories and accession number(s) can be found in the article/Supplementary material. Further inquiries can be directed to the corresponding author.

## Author contributions

DA: Data curation, Formal analysis, Investigation, Visualization, Writing – original draft, Writing – review & editing, Methodology. AM-B: Formal analysis, Investigation, Methodology, Visualization, Writing – review & editing, Software. CR-V: Formal analysis, Investigation, Methodology, Software, Visualization, Writing – review & editing, Data curation. FI: Data curation, Investigation, Methodology, Software, Visualization, Writing – review & editing, Formal analysis. MC: Investigation, Writing – review & editing, Validation. RU: Investigation, Writing – review & editing, Data curation. PC: Investigation, Writing – review & editing, Methodology. BD: Investigation, Writing – review & editing, Resources, Supervision. AM: Investigation, Supervision, Writing – review & editing, Methodology, Software, Visualization. IÑ: Investigation, Writing – review & editing, Validation. AG: Writing – review & editing, Conceptualization, Formal analysis. DJ: Conceptualization, Formal analysis, Writing – review & editing. RQ: Investigation, Project administration, Resources, Supervision, Visualization, Writing – original draft, Conceptualization, Formal analysis, Writing – review & editing, Data curation, Funding acquisition.

## Footnotes

^1^ https://www.ncbi.nlm.nih.gov/assembly/

^2^ https://github.com/dparks1134/CompareM

^3^ https://inkscape.org

^4^ http://tree.bio.ed.ac.uk/software/figtree/
