# Supplementary material for: Acidithiobacillia class members originating at sites within the Pacific Ring of Fire and other tectonically active locations and description of the novel genus ‘Igneacidithiobacillus’
Source: Front Microbiol. 2024 Apr 3;15:1360268. doi: 10.3389/fmicb.2024.1360268 (PMC11021618; doi:10.3389/fmicb.2024.1360268)
Supplement: Supplementary file 1 [file Data_Sheet_1.PDF]

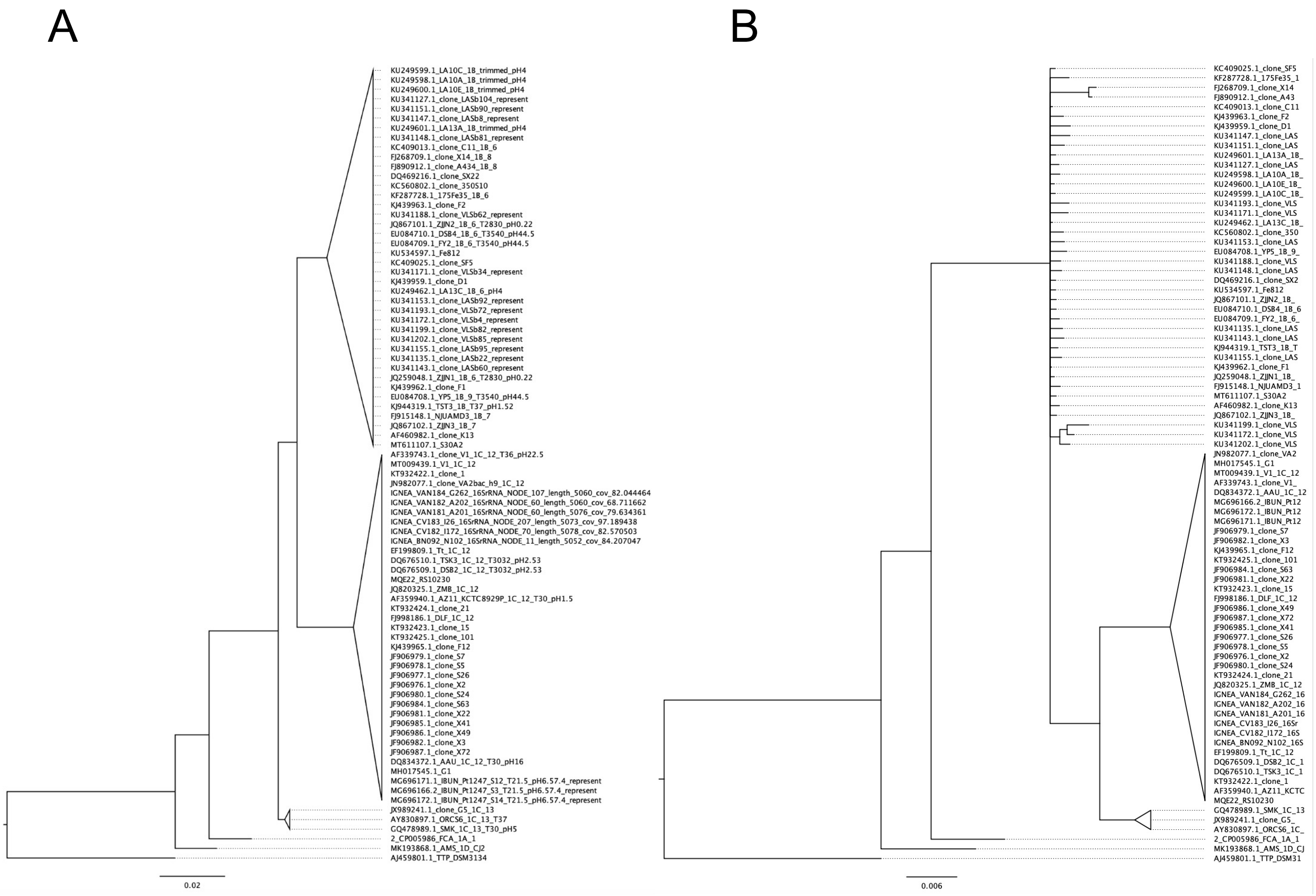

Supplementary Figure 1. Single marker based expanded phylogeny of '*Igneacidithiobacillus*' strains and sequences clones. The 16S rRNA genes retrieved form NCBI (accessed on April 2023) were aligned with MAFFT and manually refined. The alignment had 1,227 nt, 82.4% of which were constant. (A) Maximum Likelihood and (B) Bayesian Inference phylogenetic trees.

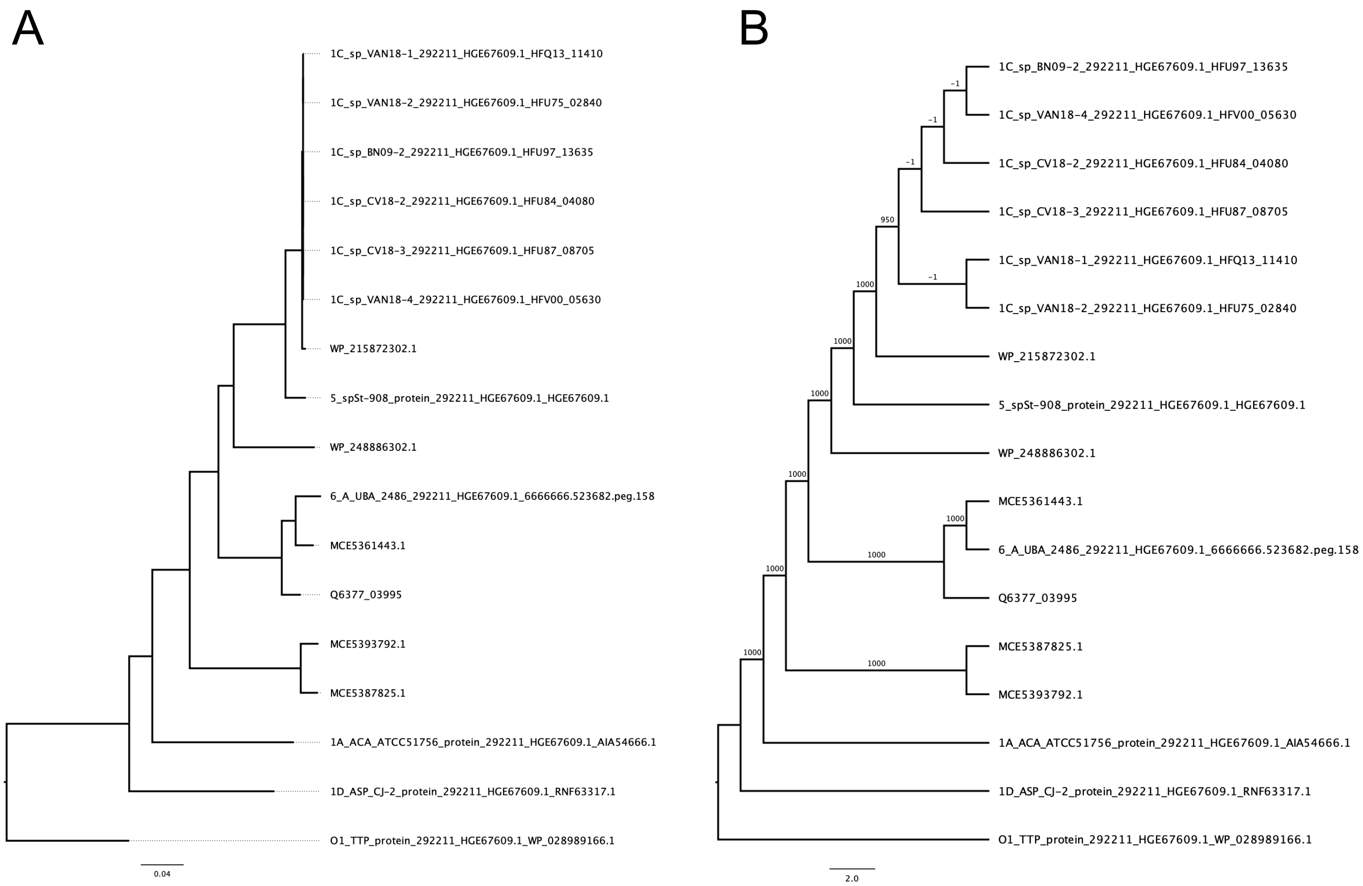

Supplementary Figure 2. Multiprotein based expanded phylogeny of '*Igneacidithiobacillus*' strains and MAGs. Phylogenetically informative housekeeping class-core marker genes used in the alignment are indicated in **Supplementary Table 6**. The final alignment contained 87 sequences and encompassed 20,789 positions (see methods). (A) Neighbor Joining and (B) Maximum Likelihood full length multiprotein phylogenetic trees of class-core marker proteins shown in **Figure 2C.**

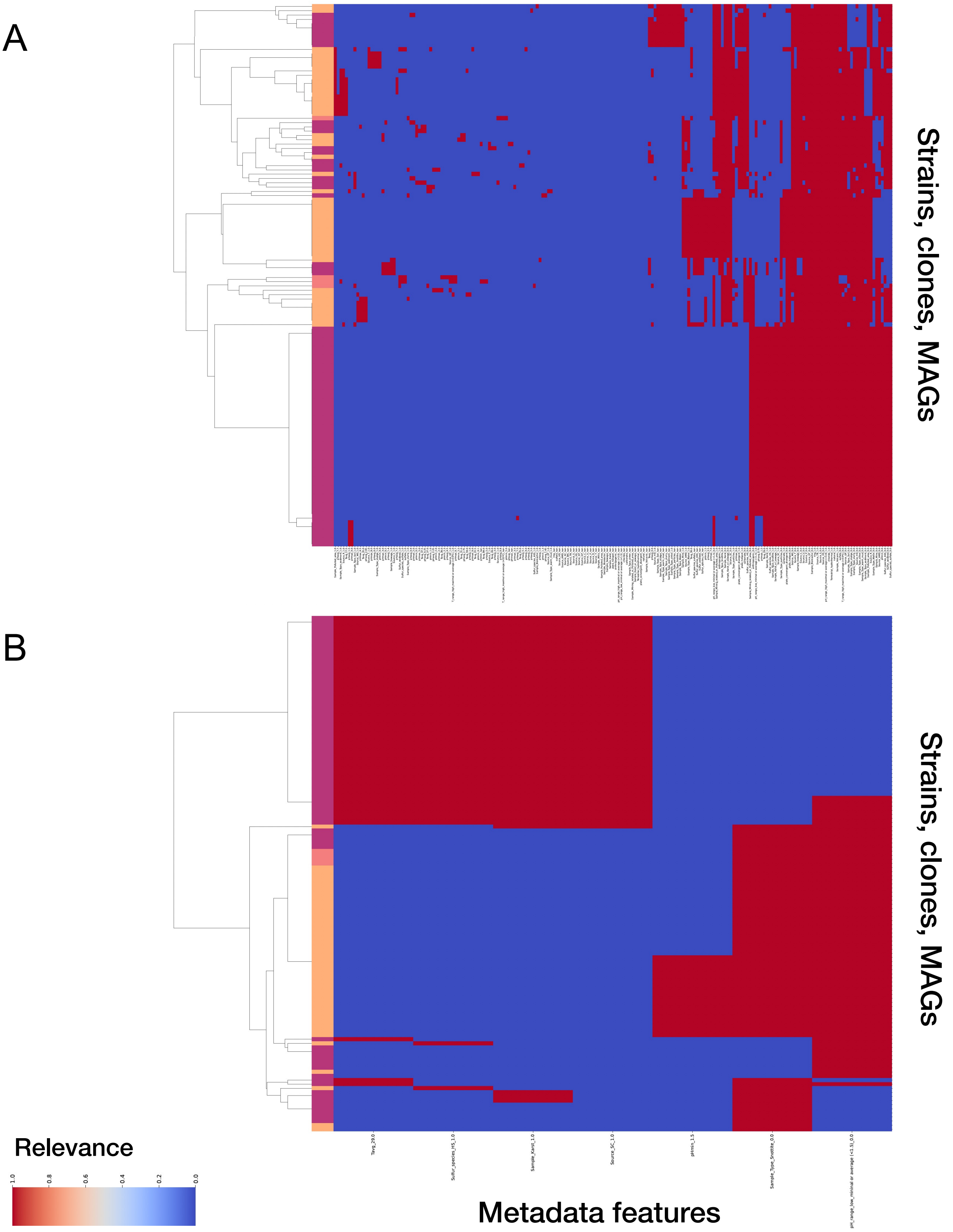

Supplementary Figure 3. Metadata based clustering analysis. Clustering based on all metadata features documented in Table 3 and Supplementary Table 2 resulted in relatively weak groupings (**A**). The most significant metadata features were ranked and narrowed down employing both the Chi-square test and a random forest (RF) algorithm. The best compromise between group separation and the number of features was obtained with the RF selecting features with relevance above 0.02. 'Sample Type Snottite', 'Sample Karst', 'pH range low minimal or low average (<1.5)', 'Source Sulfidic Cave', 'Sulfur species Hydrogen Sulfide', 'Temperature average 29.0' and 'pH min <1.5' were the most discriminative features to separate most abundantly sampled lineage representatives (**B**). Color coding of the lineages is as elsewhere in the text: '*I. copahuensis*' ; ca. I. yellowstonensis' ; '*I. siniensis*' . A number of strains/clones/MAGs with insufficient metadata did not exhibit clear separation using this approach.

A

Acronym	Test taxon (n= # genomes/MAGs)	Pro	Gly	Ala	Thr	Asn	Cys
TTP	*T. tepidarius* DSM 3134 (n=1)	997	1525	2220	817	512	160
ASM	*'A. sulfuriphilus'* CJ-2 (n=1)	984	1607	2254	819	471	180
FCA_ave	*'F. cladus'* ATCC51756 + others (n=18)	975	1551	2220	793	410	176
ITP	Ca. I. tapuoensis TVZ-G3 (n=1)	991	1489	1947	784	474	184
IWA	Ca I. waiakirensis TVZ-G4 (n=1)	950	1420	1884	763	456	170
ICH	Ca I. chanchokoensis CHCt20-39 (n=1)	945	1487	2214	800	478	184
ITA_ave	Ca. I taiwanensis UBA2486 + G2 (n=2)	916	1408	2097	745	440	178
ISI	*'I. siniensis'* S30A2 (n=1)	940	1470	2049	765	437	172
IYE	Ca. I. yellowstonensis SpSt-908.faa (n=1)	893	1451	2022	761	453	178
ICO_ave	*'I. copahuensis'* VAN18-1 + others (n=7)	922	1529	2126	781	460	184
AMA	*'A. marinus'* SH (n=1)	921	1470	2095	856	537	173
AMO	*' A. monserratiensis'* GG1-14 (n=1)	932	1481	2107	856	533	175
ASU	*' A. sulfurivorans'* RW2 (n=1)	910	1477	2105	855	560	181
ACO	*'A. concretivorus'* ATCC 19703 (n=1)	905	1480	2114	863	563	184
ATH_ave	*A. thiooxidans* ATCC 19377 (n=16)	916	1473	2103	867	567	178
AFN	*A. ferrianus* MG (n=1)	958	1569	2204	885	452	187
AFV_ave	*A. ferrivorans* DSM 22755 (n=8)	925	1556	2114	899	501	191
AFP_ave	*A. ferriphilus* DSM 100412 (n=8)	923	1570	2104	899	509	187
AFD_ave	*A. ferridurans* ATCC 33020 (n=9)	941	1575	2119	883	484	187
AFG	*A. ferruginosus* CF3 (n=1)	950	1590	2133	886	477	187
AFE_2B_ave	*A. ferrooxidans* subsp. andinus PQ 505 (n=4)	946	1589	2130	887	473	190
AFE_2A_aabe	*A. ferrooxidans* subsp. ferrooxidans ATCC_23270 (n=5)	944	1576	2125	884	474	188
	**CLASS Ave**	940	1516	2113	834	487	181

B

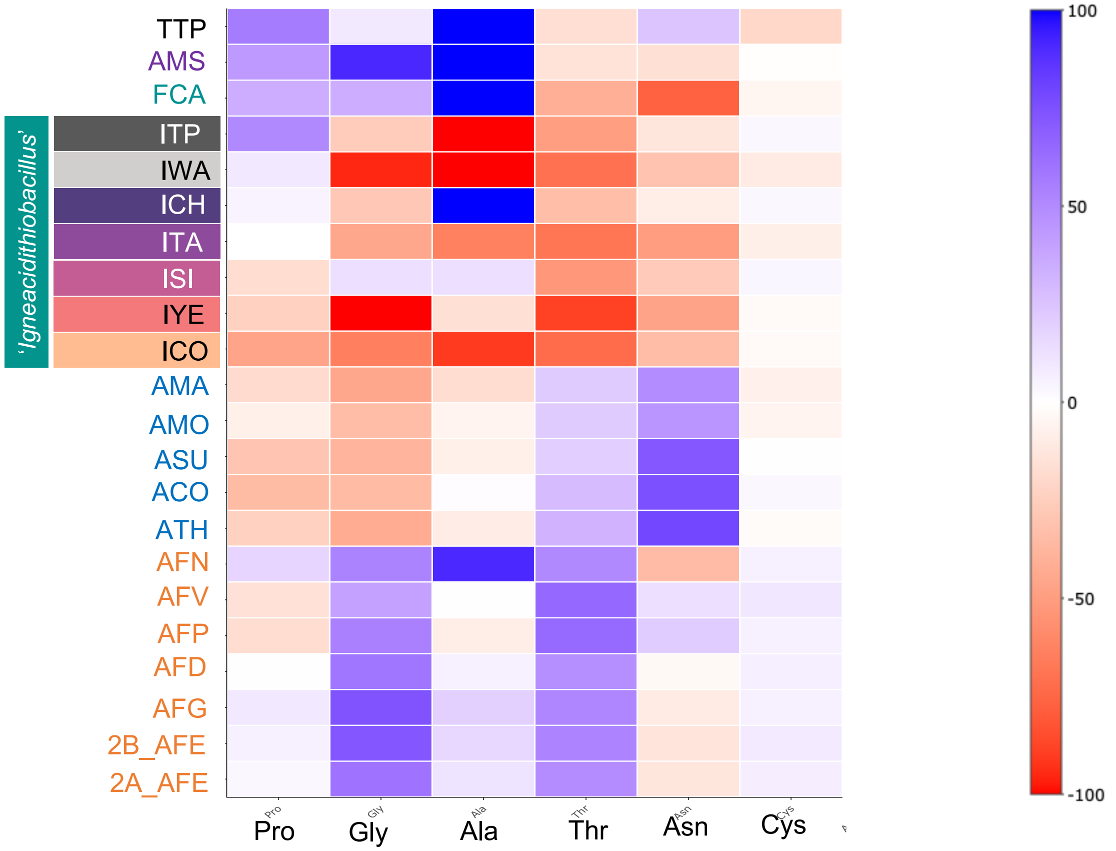

Supplementary Figure 4. Comparison of the frequency of relevant amino acids in a set of 87 orthologous core proteins of the *Acidithiobacillia* class. Residues shown were selected on the basis of their acknowledged contribution to protein stability or instability at temperatures > 50 ºC (Ahmed et al., 2022; Hait et al., 2020): Proline (Pro), Glycine (Gly), Alanine (Ala), Threonine (Thr), Asparagine (Asp) and Cysteine (Cys). (**A**) Residues count in the concatenate alignment used to construct the phylogenetic tree in Figure 2C, as a proxy for count normalization. (**B**) Count difference with respect to class average value of each residue in the same set of 87 core proteins (shown in panel A). Representatives of all genera of the class (*Thermithiobacillus*, '*Ambacidithiobacillus*', '*Fervidacidithiobacillus*', '*Igneacidithiobacillus*' and *Acidithiobacillus*) were considered in the analysis as listed in panel A.

Legends for Supplementary Multimedia Files and Tables

Supplementary Multimedia File 1. Active culture of '*I. copahuensis'* VAN18-1 grown in Umea 1X with 5% tetrathionate and 0.5% NaCl at 40ºC with cells swimming across the visual field of the phase contrast microscope (40 X). [*ICO_VAN18_1_swimming.mov*]

Supplementary Table 1. GenBank identifiers of 16S rRNA genes sequences of strains and clones used in this study (April, 2023) for phylogenetic tree reconstruction. Sequence clones are labelled "uncultured". 16S rRNA genes sequences from clade 1B/1C recovered from genomes tentatively assigned to '*Igneacidithiobacillus*' are indicated with an asterisk.

Supplementary Table 2. Metadata of strains, clones and MAGs used in this study (April, 2023). Environmental and geographical metadata associated to each strain, 16S rRNA sequence clone and/or metagenomic derived genome included in the study was recovered from published literature or public databases deposits.

Supplementary Table 3. GenBank identifiers of Fis proteins recovered from sequenced genomes and MAGs available in public databases or generated for this study, tentatively assigned to '*Igneacidithiobacillus*'.

Supplementary Table 4. Relatedness of '*Igneacidithiobacillus*' genomes and MAGs (vs. *Acidithiobacilla* class members) inferred from amino acidic (AAI) and nucleotide data using alignment-based methods (dDDH, ANIb, ANIm). Type strains are marked with a T superscript. Sequence type material is marked with a TS superscript. (**A**) Average amino acid identity (AAI) as calculated using the CompareM calculation method ([https://github.com/dparks1134/CompareM], AAICM) and and the aai.rb implementation from the Kostas Lab (Rodriguez and Konstantinidis, 2016), downloaded from github (https://github.com/lmrodriguezr/enveomics; commit signature: fae592f) and run using default parameters. Genus- and species-level AAI% cutoff thresholds used were <62-70% and >95.38, respectively (Richter and Roselló_Mora, 2009). Thresholds used for species delimitation are the following: digital DNA:DNA hybridization dDDH>70% (same genomic species, Meier-Kolthoff et al., 2013; 2014); Average Nucleotide Identity ANI > 96% (same genomic species (Pritchard et al., 2016; Richter and Roselló_Mora, 2009).

Supplementary Table 5. Supporting information on the novel taxa described in this study. Etymology for known, novel '*Igneacidithiobacillus*' species. Type material identifiers (type strain and genome accession numbers). Relevant phenotypic aspects of the described taxa including, cellular morphology, motility and lifestyle.

Legends for Supplementary Multimedia Files and Tables

Supplementary Table 6. Supporting information of the *Acidithiobacillia* class-core marker genes occurrence patterns in genomes and MAGs of ‘*Igneacidithiobacillus*’ and selected markers used in comprehensive phylogenomic analysis of the group.

Supplementary Table 7. Functional potential of ‘*Igneacidithiobacillus*’ core genes. (**A**) COGs functional gene classifications (KEGG_path rank) and their count abundance. (**B**) KEGG functional gene classifications (COG_class rank) and their count abundance. (**C**) Gene categories of interest analysed in the text including: sulfur_energy metabolism, thermotolerance, DNA repair, chaperones_ folding catalysts, acid_tolerance, osmolytes_osmotolerance, transporters, metalotolerance, amino acids biosynthesis, flagella, envelope_capsule_extracellular_matrix_components.
